# Regulating the balance between GSDMD-mediated pyroptosis and CHMP4B-dependent cell repair attenuates calcium oxalate kidney stone formation

**DOI:** 10.7150/ijbs.105415

**Published:** 2025-04-22

**Authors:** Shushuai Yang, Yuanjiong Qi, Yue Chen, Hailong Kong, Bin Han, Zhongsheng Peng, Chenglong Xu, Bohan Wang, Liqun Chen, Shiyong Qi

**Affiliations:** 1 Department of Urology, Tianjin Institute of Urology, The Second Hospital of Tianjin Medical University, Tianjin 300211, China.; 2 Department of Urology, The Second Affiliated Hospital, School of Medicine, Zhejiang University, Hangzhou 310000, China.; 3 Medical College, Academy of Medical Engineering and Translational Medicine, Tianjin University, Tianjin 300072, China.

**Keywords:** pyroptosis, cell repair, imbalance, calcium oxalate, kidney stone

## Abstract

An imbalance exists between renal tubular epithelial cells (RTECs) injury and repair in kidney stone disease, yet the underlying mechanism remains largely unclear. Here, we found that gasdermin D (GSDMD)-mediated pyroptosis occurred in both patients and mice with calcium oxalate (CaOx) nephrolithiasis, and the expression levels of NOD-like receptor protein 3 (NLRP3) and GSDMD were associated with the severity of kidney stones. Deficiency of GSDMD alleviated renal tubule damage and inflammatory response, ultimately inhibiting renal injury and crystal deposition. Additionally, we found that charged multivesicular body protein 4B (CHMP4B)-dependent cell repair was activated during pyroptosis of RTECs; however, the enhancement was insufficient to offset the damage. Importantly, Ca^2+^ fluxes during pyroptosis induce activation of the CHMP4B-dependent cell repair machinery. Overexpression of CHMP4B attenuates cell death and reduces the severity of kidney stones. Notably, combining the overexpression of CHMP4B with a GSDMD inhibitor demonstrates heightened efficacy in ameliorating kidney damage and crystal deposition induced by glyoxylate (Gly). Taken together, these results highlight the imbalance between GSDMD-mediated pyroptosis and CHMP4B-dependent cell repair as a driver for CaOx kidney stone formation. Our findings provide new insights and potential therapeutic targets for CaOx kidney stones.

## Introduction

Kidney stone disease (nephrolithiasis, urinary stones, and renal calculi) is a common disease of the urinary system that affects a significant portion of the world's population, and the prevalence rates seem to be rising in nearly all countries 1, 2. Kidney stones exhibit diverse compositions, with approximately 75% of cases involving calcium oxalate (CaOx) stones 3. The pathophysiology of kidney stone formation remains complex and multifactorial. It is currently known that tubular epithelial cell injury plays an extremely critical role in stone formation by enhancing the cell-crystal adhesion to renal tubular epithelial cells (RTECs) and facilitating the crystallization process, but the specific molecular mechanisms have not been fully elucidated 4.

During kidney stone formation, renal tubular epithelial cell injury involves multiple mechanisms of programmed cell death, including apoptosis, necroptosis, pyroptosis, ferroptosis, and autophagy, et al. These pathways can be triggered by various factors, such as oxidative stress, inflammation, and mitochondrial dysfunction, ultimately leading to cell death and impaired repair response 5-9. Recent studies have provided convincing evidence that pyroptosis is involved in the formation and development of CaOx kidney stones 10-12. Pyroptosis, an inflammatory form of programmed cell death characterized by cell swelling, cell membrane disruption, and release of cytoplasmic content, is associated with the development of kidney diseases 13, 14. In response to specific cell death signals, such as those mediated by the NOD-like receptor protein 3 (NLRP3) inflammasome, caspase-1/4/5/11 enzymes cleave Gasdermin D (GSDMD), activating it into the N-terminal fragment of GSDMD (GSDMD-N). The GSDMD-N inserts into cellular membranes to mediate the formation of membrane pores 15, 16. This process leads to the release of cytokines, alarmins, and damage-associated molecular patterns (DAMPs), ultimately resulting in cell membrane rupture and triggering cell death 17. Pyroptosis is a type of cell death characterised by inflammation that effectively activates the immune system, and is distinct from apoptosis, which is typically regulated through intracellular signaling pathways, leading to the programmed death of cells, usually without eliciting an inflammatory response 18. Ferroptosis is characterized by the rupture of the cell membrane due to the accumulation of lipid peroxides. In contrast, pyroptosis involves the formation of pores in the cell membrane through inflammatory signaling pathways and the activation of gasdermin proteins, ultimately leading to the release of cellular contents 19, 20. Thus, pyroptosis has a unique morphology and mechanism compared to other cell death types and can trigger an inflammatory response 21.

Interestingly, damaged cells undergo rapid repair where cell membrane is quickly resealed, allowing the cells to resume normal function 22. The maintenance of cellular structure and function relies on a delicate balance between cellular damage and repair via multiple mechanisms 23, 24. Mammals are capable of repairing cellular damage through a variety of mechanisms to maintain normal cellular structure and function, in which the endosomal sorting complex required for transport (ESCRT)-mediated cellular repair plays a critical role in maintaining life activities 25-28. Substantial evidence indicated that ESCRT-dependent membrane repair negatively regulates pyroptosis, with charged multivesicular body protein 4B (CHMP4B) being involved in the repair process 27-29. CHMP4B is a crucial component of the ESCRT-III complex, playing a significant role in cell membrane repair and cytokinesis 30. Additionally, CHMP4B is instrumental in autophagy, particularly in the elimination of damaged mitochondria 31. During this process, CHMP4B is recruited to the stage of autophagosome formation, thereby facilitating the closure and functionality of autophagosomes 32. Furthermore, the regulation of CHMP4B is linked to cellular responses to oxidative stress and various forms of injury, which further emphasises its multiple roles in cell repair 33. In the context of cell membrane damage, the ESCRT complex facilitates membrane remodeling and repair through the polymerization of CHMP4B. This process involves the polymerization of CHMP4B along with interactions with other essential ESCRT proteins that are crucial for membrane cleavage and remodeling 34. Notably, a recent study confirmed that there is a significant association between CHMP4B and kidney disease, and its abnormal function or altered expression may be involved in the occurrence of renal injury 35. However, the specific role and detailed mechanism of CHMP4B in CaOx kidney stone disease have yet to be known.

Previous studies on the mechanisms of renal stone formation have primarily focused on renal tubular epithelial cell injury, with few studies addressing the maintenance of the balance between cell damage and repair to inhibit stone formation. Therefore, we propose a novel hypothesis that there is a balance between GSDMD-mediated pyroptosis and CHMP4B-dependent cell repair during CaOx kidney stone formation, and that regulating the balance between damage and repair contributes to the maintenance of cellular homeostasis, ultimately inhibiting stone formation. In our study, we investigated the equilibrium mechanism of the GSDMD/CHMP4B signaling axis to provide new therapeutic targets for CaOx kidney stones.

## Materials and Methods

### Clinical kidney specimens and blood sample preparation

Human kidney samples from patients with kidney cancer and kidney stones were obtained from the Second Hospital of Tianjin Medical University. Peripheral blood was collected from 20 patients with renal stones and 20 healthy donors matched for gender and age distribution. The whole blood RNA was isolated using the Blood Total RNA Isolation Kit (RE-04013, Foregene, China) according to the manufacturer's protocol and previous reports 36, 37. This study was approved by the Institutional Ethics Committee of the Second Hospital of Tianjin Medical University (ethical approval number: KY2024K242), and informed consent was obtained from all participating patients. Clinical characteristics of the human subjects are presented in Table S1.

### Animal experiments

The animal experiments in this study obtained approval from the Animal Ethics Committee at the Second Hospital of Tianjin Medical University (Tianjin, China; approval no. KY2022K103). All the procedures complied with the international guidelines for the use of experimental animals. Male C57BL/6 mice aged 6 to 8 weeks old were purchased from Vitonlihua (Beijing, China). GSDMD knockout (GSDMD-KO) mice were acquired from Chen's Laboratory at Tianjin University. The GSDMD-KO mice were generated through intercrossing heterozygous GSDMD+/‑ mice, with genotyping performed to confirm the genotype. All mice were fed standard food and water, and housed in a specific pathogen-free facility under a 12-h light/dark cycle. For the animal experiments, each group consisted of 6 mice.

C57BL/6 male mice were used for the animal experiments, and experimental grouping was performed as follows:

The control group (Control group): An equal volume of saline was intraperitoneally injected once daily for 7 consecutive days.

The glyoxylate group (Gly group): The CaOx nephrocalcinosis mouse model was established according to our previous and the reported methods 38, 39. Briefly, mice received intraperitoneal injection with glyoxylate (100 mg/kg/d; 563-96-2; Macklin) for 7 consecutive days.

The glyoxylate + necrosulfonamide (NSA) group (Gly + NSA group): The mice received a daily intraperitoneal injection of glyoxylate (100 mg/kg/d). Subsequently, mice were treated daily with NSA (ab143839; Abcam) via intraperitoneal injection at a dose of 50 mg/kg for 7 consecutive days.

The glyoxylate + adeno-associated virus (AAV)-CHMP4B group (Gly + AAV-CHMP4B group): To discuss the effect of CHMP4B, C57BL/6 male mice were injected with AAV-FLEX-CHMP4B (80 mg/kg, 100 µL) via tail vein. Dosages of AAV-FLEX-CHMP4B were based on the manufacturer's instructions. Two weeks later, all mice received intraperitoneal injection of glyoxylate (100 mg/kg/d) for 7 days.

The glyoxylate + NSA + AAV-CHMP4B group (Gly + NSA + AAV-CHMP4B group): Similar to the glyoxylate + AAV-CHMP4B group except that, the difference is that the glyoxylate was replaced with glyoxylate and NSA.

The specific sequences of AAV-FLEX-CHMP4B are listed below:

ATGTCGGTGTTCGGGAAGCTGTTCGGGGCTGGAGGGGGTAAGGCGGGCAAGGGCGGCCCGACCCCCCAGGAGGCCATCCAGCGGCTTCGGGACACGGAGGAGATGTTAAGCAAGAAGCAGGAGTTCCTGGAGAAGAAAATCGAACAGGAGCTGACGGCTGCCAAGAAGCACGGCACCAAAAATAAACGCGCCGCCCTGCAGGCTCTGAAGCGCAAGAAGAGGTATGAGAAGCAGCTGGCACAAATTGATGGCACCCTGTCAACCATCGAGTTCCAGCGGGAGGCCCTAGAGAACGCCAACACCAACACGGAGGTGCTCAAGAACATGGGCTATGCCGCCAAGGCCATGAAGGCTGCCCACGACAACATGGACATTGATAAGGTGGATGAGTTAATGCAGGACATTGCTGACCAGCAAGAACTTGCAGAGGAGATTTCCACAGCTATCTCCAAACCTGTGGGCTTTGGAGAAGAGTTCGACGAGGATGAGCTCATGGCAGAGTTGGAGGAACTTGAACAAGAGGAGTTGGACAAGAATTTGTTGGAGATCAGTGGGCCCGAAACAGTCCCTCTACCAAATGTCCCCTCCGTAGCCCTACCATCCAAACCCGCCAAGAAGAAGGAAGAGGAAGATGACGACATGAAGGAATTGGAGAACTGGGCCGGATCCATGTAA.

Wild-type mice (WT) or GSDMD-KO mice were used for the animal experiments, and experimental grouping was performed as follows:

The wild-type group (WT group): WT mice received intraperitoneal injection with an equal volume of saline for 7 consecutive days.

The GSDMD-KO group (GSDMD-KO group): GSDMD-KO mice received intraperitoneal injection with an equal volume of saline for 7 consecutive days.

The wild-type + glyoxylate group (WT + Gly group): WT mice received intraperitoneal injection with glyoxylate (100 mg/kg/d) for 7 consecutive days.

The GSDMD knockout + glyoxylate group (GSDMD-KO + Gly group): GSDMD-KO mice received intraperitoneal injection with glyoxylate (100 mg/kg/d) for 7 consecutive days.

### Cell culture and intervention

The human renal tubular epithelial cell line (HK-2) was purchased from Shanghai Institutes for Biological Sciences (Shanghai, China). HK-2 cells were cultured using DMEM medium (MA0212, Meilunbio) supplemented with 10% FBS (FSD500; ExCell) and 1% penicillin-streptomycin (MA0110, Meilunbio). The cells were incubated in a 5% CO2 incubator at 37 °C. Subsequently, the negative control (NC) group was exposed to a complete medium for 24 h, the oxalate (Ox) group was treated to 0.8 mmol/L oxalate, the Ox + NSA group was treated with 0.8 mmol/L oxalate and 15 μmol/L NSA for 24 h, and the Ox + si-GSDMD group was pretransfected with GSDMD siRNA and was treated with 0.8 mmol/L oxalate for 24 h. The Ox + oe-CHMP4B group was pretransfected with CHMP4B plasmids and was treated with 0.8 mmol/L oxalate for 24 h. The Ox + NSA + oe-CHMP4B group was pretransfected with CHMP4B plasmids and was treated with 0.8 mmol/L oxalate and 15 μmol/L NSA for 24 h.

### Von Kossa staining

Renal crystal deposition was detected by Von Kossa staining. Kidney retention, embedding, section, dewaxing and cleaning. The slices were placed in silver nitrate staining solution, irradiated by ultraviolet light for 10 min, washed, retained and sealed. Finally, the records were observed under a microscope. Image J was used for semi-quantitative analysis.

### TUNEL assay

According to the manufacturer's instructions, the one-step TUNEL cell apoptosis detection kit (MA0224, meilunbio) was utilized to identify cellular apoptosis. Cell samples were fixed with 4% PFA for 20 min at room temperature (RT), followed by washing. Subsequently, dilute Proteinase K (2 mg/ml) in PBS at a ratio of 1:100, achieving a final concentration of 20 µg/ml. Incubate this mixture at RT for 10 min. For paraffin-embedded renal tissues, the samples underwent a series of preparatory steps including deparaffinization, hydration, washing, and permeabilization. Next, introduce the TUNEL working solution (prepared immediately before use) and incubate at 37 °C in darkness for 60 min. Afterward, perform three washes with PBS. Then, apply anti-fade mounting medium with DAPI (MA0236, meilunbio), and proceed to observe and enumerate TUNEL-positive cells were observed and counted under a fluorescence microscope (Nikon, Tokyo, Japan).

### Cell-crystal adhesion

Calcium oxalate monohydrate (COM) crystals were synthesized using sodium oxalate (D6830, Solarbio) and calcium chloride (C7250, Solarbio), followed by labeling with fluorescein isothiocyanate (FITC) as previously described 40. The adhesion of COM crystals to HK-2 cells was evaluated using a fluorescence microscopy. Images were acquired and subsequently analyzed with Image J software. The area occupied by FITC-labeled COM crystals was quantified and expressed as a percentage of the total imaging area.

### RNA sequencing and analysis

The kidney tissues of control mice, Gly-treated mice and Gly-treated AAV-FLEX-CHMP4B-infected mice were collected. A minimum of three biological replicates for each group were used for RNA-Seq. Total RNA was extracted from 30-50 mg kidney tissue samples, and analysed by Bioanalyzer 2100 and RNA 6000 Nano Kit (Agilent, USA). RNA samples with RIN number > 7.0 were used to construct a sequencing library. The sequencing library was employed for transcriptome sequencing, utilizing the Illumina NovaSeq 6000 system (Lianchuan Biotechnology, Hangzhou, China). The differential gene expression (DEG) analysis was performed using the DESeq2 software and padj < 0.05 and |log2(foldchange)| ≥ 1 were set as the threshold for significantly differential expression. Finally, we utilized bioinformatics tools on a cloud platform (link: https://www.omicstudio.cn/home) to analyze the raw data.

### siRNA transfection and plasmid transfection

For siRNA transfection:

Stranded siRNA targeting *NLRP3*, *GSDMD* or *CHMP4B* (si-*NLRP3*, si-*GSDMD*, or si-*CHMP4B*) and the negative control siRNA (si-*NC*) were purchased from GenePharma (Shanghai, China). HK‑2 cells were transfected using siRNA mixed with Lipofectamine 3000 reagent (Invitrogen, USA) in accordance with the manufacturer's protocol. The transfection efficiency was determined by quantitative real-time PCR (qRT-PCR) and Western blot at 48-72 h after transfection. The sequences of siRNAs targeting *NLRP3*, *GSDMD*, and *CHMP4B* are shown below.

si-*NLRP3* (human): sense GCUGCUGAAAUGGAUUGAATT, antisense UUCAAUCCAUUUCAGCAGCTT.

si-*GSDMD* (human): sense GAGCUUCCACUUCUACGAUTT, antisense AUCGUAGAAGUGGAAGCUCTT.

si-*CHMP4B* (human): sense GGCGGAAUUAGAAGAACUATT, antisense UAGUUCUUCUAAUUCCGCCTT.

For plasmid transfection:

The plasmids for *CHMP4B* overexpression (oe-*CHMP4B*) and the negative control (oe-*NC*) were procured from GenePharma (Shanghai, China). The oe-*CHMP4B* or oe-*NC* was transfected into HK-2 cells with Lipofectamine 3000 reagent (Invitrogen, USA) in accordance with the manufacturer's protocol. The transfection efficiency was determined by qRT-PCR and Western blot at 48-72 h after transfection. The plasmid for *CHMP4B* overexpression was constructed as follows.

ATGTCGGTGTTCGGGAAGCTGTTCGGGGCTGGAGGGGGTAAGGCCGGCAAGGGCGGCCCGACCCCCCAGGAGGCCATCCAGCGGCTGCGGGACACGGAAGAGATGTTAAGCAAGAAACAGGAGTTCCTGGAGAAGAAAATCGAGCAGGAGCTGACGGCCGCCAAGAAGCACGGCACCAAAAACAAGCGCGCGGCCCTCCAGGCACTGAAGCGTAAGAAGAGGTATGAGAAGCAGCTGGCGCAGATCGACGGCACATTATCAACCATCGAGTTCCAGCGGGAGGCCCTGGAGAATGCCAACACCAACACCGAGGTGCTCAAGAACATGGGCTATGCCGCCAAGGCCATGAAGGCGGCCCATGACAACATGGACATCGATAAAGTTGATGAGTTAATGCAGGACATTGCTGACCAGCAAGAACTTGCAGAGGAGATTTCAACAGCAATTTCGAAACCTGTAGGGTTTGGAGAAGAGTTTGACGAGGATGAGCTCATGGCGGAATTAGAAGAACTAGAACAGGAGGAACTAGACAAGAATTTGCTGGAAATCAGTGGACCCGAAACAGTCCCTCTACCAAATGTTCCCTCTATAGCCCTACCATCAAAACCCGCCAAGAAGAAAGAAGAGGAGGACGACGACATGAAGGAATTGGAGAACTGGGCTGGATCCATGTAA.

### Western blot analysis

The total proteins of the kidney tissues and HK-2 cells were extracted by radioim-munoprecipitation assay (RIPA) (P0013B, Beyotime) supplemented with 1% PMSF (P0100, Solarbio) and 1% phosphatase inhibitors (GK10012, Glpbio). Protein concentrations were measured with a BCA Protein Quantification Kit (ZJ102, Yamei, China). Protein samples was boiled at 95 °C for 5 min with 4× SDS-PAGE loading buffer (P1016, Solarbio) and then separated by sodium dodecyl sulfate-poly-acrylamide gel electrophoresis (SDS-PAGE). The separated proteins were transferred to polyvinylidene fluoride (PVDF) membranes (ISEQ0001, Solarbio), which were blocked with 5% skim milk (D8340, Solarbio) at RT for 1 h. The membranes were washed with TBST (3 × 5 min) and incubated with the primary antibodies at 4 ℃ overnight. Then, the membranes were rewashed with TBST (3 × 15 min), soaked in HRP-conjugated secondary antibodies, and incubated at RT for 1 h. After washing with TBST (3 × 15 min), the detection was performed with an ECL detection kit (E-IR-R308, Elabscience). The chemifluorescence was detected by Tanon-5200 Imaging system (Shanghai, China). Image J software was used to calculate the grayscale values of the proteins, and GAPDH or β-actin was used as a reference for normalization. The antibodies used are detailed in Table S2.

### RNA extraction and quantitative real-time PCR

Total RNA of kidney tissues or HK-2 cells was extracted by Trizol Substitute (R1100, Solarbio, China). The Strand cDNA Synthesis SuperMix (11141ES60, Yeasen, China) was used to reverse transcribe RNA into cDNA, and then SYBR Green Master Mix (11201ES08, Yeasen, China) was used for qRT-PCR experiments according to the manufacturer's instructions. GAPDH was utilized as a reference gene, and all PCR experiments were performed in triplicate. The primer sequences are detailed in Table S3.

### Immunofluorescence staining

HK-2 cells were washed 3 times with PBS, fixed with 4% paraformaldehyde (PFA) at 37 °C for 15 min and permeabilized with 0.3% Triton X-100 at RT for 15 min. Then, the cells were blocked with 10% normal goat serum for 1 h before incubation with the primary antibodies (antibody information shown in Table S2) at 4 °C for 8 h. After extensive washes with PBS, fluorescently conjugated secondary antibodies were applied at 1:500 dilution for 1 h at RT. The secondary antibodies used were as follows: Alexa Fluor 594 goat anti-rabbit (A-11012, Invitrogen), Alexa Fluor 488 goat anti-mouse (A-11001, Invitrogen). The nucleus was counterstained with DAPI (C0065, Solarbio) for 5 min. Cells were observed using a confocal microscope (Nikon, Japan). Analyses of fluorescence intensities and colocalization were performed using Image J software.

### Immunohistochemistry

4% PFA-fixed kidney tissues were embedded in paraffin, and sections (10 μm) were deparaffinized and rehydrated. The tissue sections were incubated in pH 9.0, 1 x Tris-EDTA buffer (C1038, Solarbio) at 95 °C for 15 min for antigen repair. After blocking endogenous peroxidase, sections were incubated with primary antibodies at 4 °C overnight. Then, PV-9000 two-step immunohistochemical kit (PV-9000, ZSGB-BIO, China) was used according to the manufacturers protocol. Sections were stained with a DAB kit (DA1010, Solarbio), and hematoxylin (G1080, Solarbio) was used to stain the nuclei. Stained sections were imaged using a Nikon Eclipse Ci microscopy (Tokyo, Japan). Primary antibodies listed in Table S2.

### Cell viability assay

Cell viability was determined with cell counting kit-8 assay (CCK-8, GK10001, Glpbio) according to the manufacturer's instructions. HK-2 cells were incubated with the CCK8 reaction buffer for 1 h under different treatment conditions. The light absorbance was measured at 450 nm using the 800TS Absorbance Reader (BIOTEK, USA). The cell viability (%) was calculated by comparing the OD value to that of the control group.

### Enzyme-linked immunosorbent assay (ELISA)

Serum concentrations of IL-1β and IL-18 were measured using ELISA with commercial kits (E-EL-M0037, E-EL-M0730, Elabscience), in accordance with the manufacturer's instructions. In brief, mouse serum was added to a 96-well ELISA plate and subsequently incubated with primary and secondary antibodies. The standard curves were generated by IL-1β or IL-18 standards. The optical absorbance was quantified using the 800TS Absorbance Reader (BIOTEK, USA) at a wavelength of 450 nm, with the results reported in picograms per milliliter (pg/mL).

### Intracellular Ca^2+^ level detection and live-cell confocal imaging

The Fluo-4 Calcium Assay Kit (S1061S, Beyotime) is employed to quantify intracellular Ca^2+^ levels. Following the manufacturer's protocol, an appropriate volume of Fluo-4 staining solution should be prepared and homogenized. This staining solution is then added to the cells in the indicated volume. The cells are subsequently incubated in the dark at 37 ºC for 30 min. Finally, alterations in intracellular Ca^2+^ levels are measured using either a fluorescence microscope or a flow cytometer.

The Cell Plasma Membrane Staining Kit with Dil (C1991S, Beyotime) is employed for the red fluorescent staining and labeling of cellular membranes. During the procedure, HK-2 cells are seeded onto cell culture dishes, and the cell culture medium is aspirated. The cells are then washed twice with PBS, followed by the addition of an appropriate volume of the cell membrane staining working solution. The cells are incubated in the dark at 37 °C for a period ranging from 5 to 20 min. After washing 2-3 times with PBS, the nuclei are then counterstained with Hoechst 33342 (C0030, Solarbio), followed by the addition of pre-warmed cell culture medium at 37 °C. Finally, the cells were observed using a high-resolution live cell imaging system.

### Calcein-AM/PI staining

The Calcein-AM/PI Double Stain Kit (E-CK-A354, Elabscience) was used to stain the live/dead cell. HK-2 cells were cultured in 24-well plates. After 24 h of treatment, the medium solution in the wells was removed. Prepare the staining solution according to the manufacturer's instructions, and add 200 μL of staining solution to each well of the 24-well plate. After 20 min, the staining effect was observed by a fluorescence microscope (Calcein-AM is green fluorescence, Ex/Em=494nm/517nm; PI is red fluorescence, Ex/Em=535nm/617nm).

### Transmission electron microscopy (TEM)

The ultrastructure of organelles was examined with a transmission electron microscope (Hitachi, HT7700, Japan) following standard procedures. Kidney tissues were fixed in electron microscopy fixation fluid (G1102, Servicebio) to preserve cellular integrity. Dehydrate the sample to remove water content. Stain thin sections with uranyl acetate and lead citrate. Observe stained samples using a transmission electron microscope to examine the morphology and size of organelles such as the endoplasmic reticulum, mitochondria, autophagosomes, golgi apparatus and capture digital images.

### Statistical analysis

SPSS 26.0 statistical software was used for statistical analysis. Normally experimental data were expressed as means ± standard error of the mean (SEM) and analyzed using GraphPad Prism 8.4.0. software. Student's t-test was employed for comparisons between two groups, and one-way analysis of variance (ANOVA) was used for groups of three or more. Statistical significance was defined as *P < 0.05, **P < 0.01, ***P < 0.001, and ns represented non-significance. *In vitro* and *in vivo* experiments were independently repeated at least three times.

## Results

### GSDMD-mediated pyroptosis was significantly activated in CaOx kidney stone patients and mice

**Figure 1:**
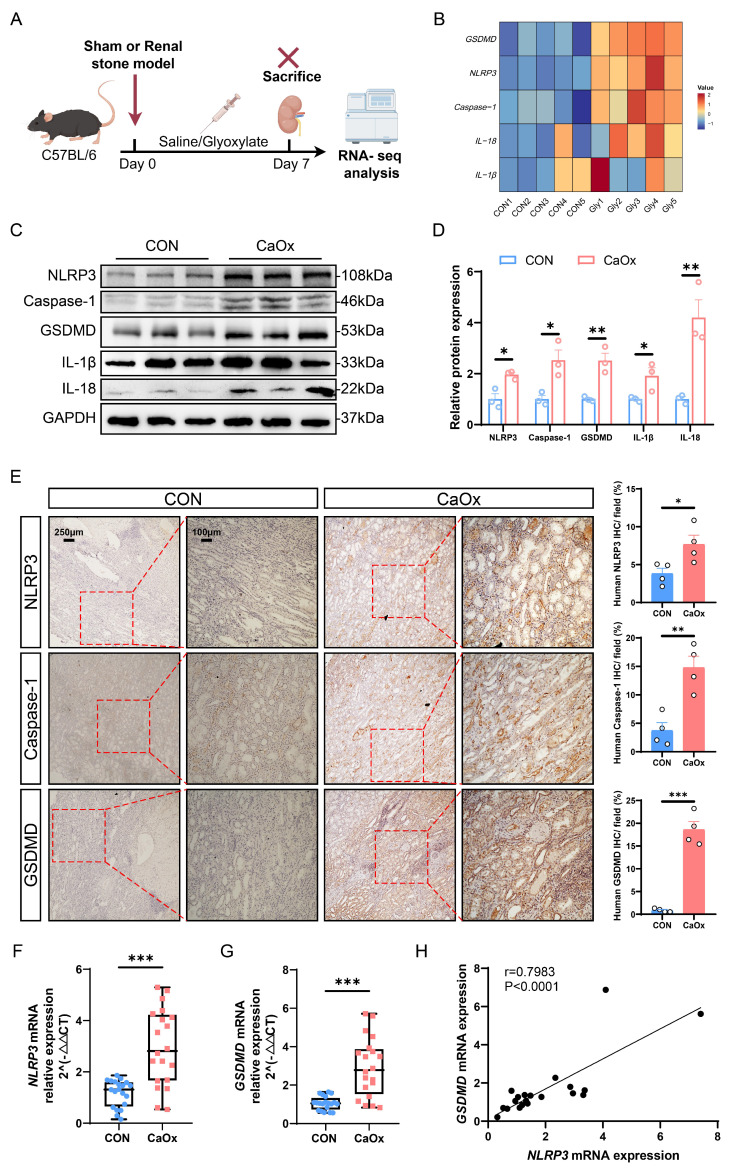
** Pyroptosis-related genes were significantly upregulated in kidney tissues from stone patients and mice. A** Experimental schema for establishing animal model of kidney stones in C57BL/6 mice. The experimental mice were induced through intraperitoneal injection of Gly (100 mg/kg/d), and the kidney tissues were obtained after treatment for 7 days. **B** The heat map showed the mRNA changes of *NLRP3*, *Caspase-1*, *GSDMD*, *IL-1β*, and *IL-18* in kidney tissues (n = 5). **C, D** Western blot images (**C**) and quantitative plots (**D**) of NLRP3, Caspase-1, GSDMD, IL-1β, and IL-18 expression in kidney tissues from normal people and patients with kidney stones (n = 3). **E** Representative images and statistical graphs for immunohistochemical (IHC) staining of NLRP3, Caspase-1, and GSDMD in kidney tissues from normal people and patients with kidney stones (n = 4). **F, G** Relative mRNA expression of *NLRP3* and *GSDMD* was assessed by qRT-PCR in 20 normal people and 20 patients with kidney stones (n = 20). **H** The linear regression analysis of the relevance between *NLRP3* mRNA expression and *GSDMD* mRNA expression (r = 0.7983, P < 0.0001, n = 20). Data are presented as mean ± SEM. *P < 0.05, **P < 0.01, ***P < 0.001.

To explore the genes and signaling pathways associated with kidney stone formation, we established a mouse model of CaOx nephrolithiasis by administering intraperitoneal injections of glyoxylate (Gly), which is a commonly used kidney stone model, and collected kidney tissues for genomics analysis (Fig. 1A). RNA-seq analysis indicated the significant enrichment of cell adhesion, inflammatory response, chemokine signaling pathway, and NOD-like receptor signaling pathway was significantly activated in the kidneys with stones (Fig. S1A-S1B). Studies have demonstrated that the NLRP3 inflammasome plays a crucial role in the process of pyroptosis, which activates Caspase-1 upon sensing a stimulus signal, leading to the cleavage and activation of GSDMD 41. Once cleaved and activated by Caspase-1, GSDMD-N binds to cell membrane, causing inflammatory cell rupture and the release of intracellular pro-inflammatory factors such as interleukin-1β (IL-1β) and interleukin-18 (IL-18) 42. As depicted in Fig. 1B, the heatmap analysis revealed notable upregulation of genes associated with pyroptosis, including *GSDMD*, *NLRP3*, *Caspase-1*, *IL-18*, and *IL-1β*. Indeed, calcium oxalate mainly promoted the mRNA expression of genes associated with pyroptosis, although it also induced the mRNA expression of ferroptosis-, apoptosis- or necrosis-related genes to some extent (Fig. S1C). The morphology of renal tubular epithelial cells was analyzed by transmission electron microscopy (TEM). The results showed that the renal tubular epithelial cells in the stone group exhibited clear signs of pyroptosis, characterized by loss of plasma membrane integrity, cell swelling, swollen and damaged organelles, and cytoplasmic vacuolization (Fig. S1D). Furthermore, to further clarify the type of cell death in renal stone disease, we exposed HK-2 cells to Ox to construct a cell model of CaOx stone. Subsequently, we carried out cell viability assays to investigate the effects of pyroptosis, ferroptosis, necrosis, and apoptosis inhibitors on Ox-induced cell death. Notably, pretreatment with NSA, a pyroptosis inhibitor, significantly enhanced the viability of HK-2 cells in the Ox group, whereas pretreatment with ferrostatin-1 (a ferroptosis inhibitor), necrostatin-1 (a necroptosis inhibitor), or Z-VAD-FMK (an apoptosis inhibitor) did not yield similar results (Fig. S1E). This suggests that pyroptosis is the major form of cell death in CaOx kidney stones. To further investigate the mechanism of cellular damage during kidney stone formation, we focused on NLRP3/GSDMD-mediated pyroptosis.

Subsequently, we evaluated the expression levels of pyroptosis-related genes in kidney tissues from both normal individuals and patients with CaOx stones. Western blotting analysis demonstrated markedly increased expressions of NLRP3, Caspase-1, GSDMD, IL-1β, and IL-18 in kidneys from patients with stones (Fig. 1C, D), and similar outcomes were observed in immunohistochemistry (Fig. 1E and S1F). Furthermore, to investigate the role of NLRP3/GSDMD-mediated pyroptosis in renal stone formation, we conducted qRT-PCR assays on blood samples (20 normal individuals vs. 20 kidney stone patients). Quantitative analysis revealed significantly higher mRNA expression of *NLRP3*, *GSDMD*, and crystal adhesion factor (cluster of differentiation 44, *CD44*) in kidney stone samples than in normal samples (Fig. 1F, G and S1G). Additionally, linear regression analysis confirmed a positive correlation between the mRNA expression levels of *NLRP3* and *GSDMD* with that of *CD44*, respectively (Fig. S1H-S1I). More importantly, *NLRP3* mRNA expression was positively correlated with *GSDMD* mRNA expression (Fig. 1H). All these results suggest that the NLRP3/GSDMD axis is activated in the kidneys of patients and mice with renal calculi, indicating the potential involvement of pyroptosis in the pathogenesis of CaOx kidney stones.

### GSDMD deficiency attenuated CaOx crystal deposition and kidney damage by inhibiting pyroptosis *in vivo*

**Figure 2:**
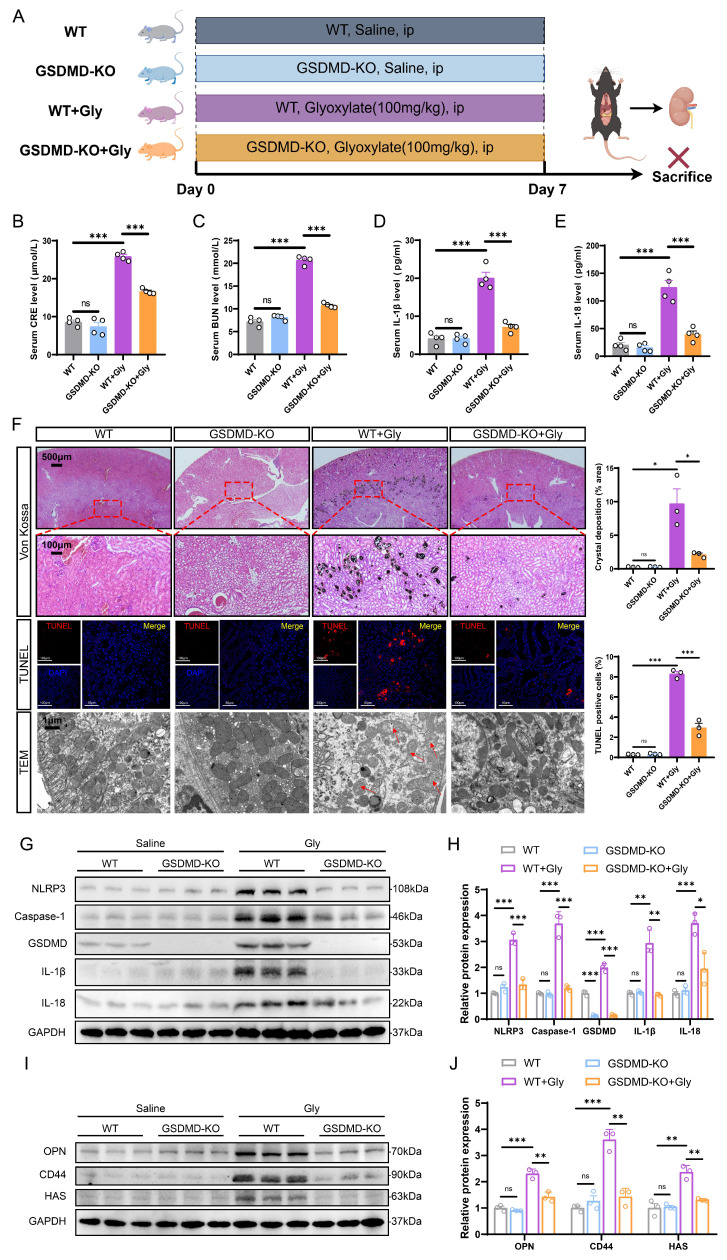
** GSDMD deficiency ameliorated kidney injury and crystal deposition caused by pyroptosis in the stone model. A** Experimental outline for establishing the kidney stone model in WT or GSDMD-KO mice. Kidneys were harvested from WT and GSDMD-KO mice after 7 days of intraperitoneal glyoxylate injection. ip, intraperitoneal. **B, C** The contents of CRE (**B**) and BUN (**C**) in the serum of each group of mice (n = 4). **D, E** The contents of IL-1β (**D**) and IL-18 (**E**) in the serum of each group of mice (n = 4). **F** Representative images and quantitative plots of Von Kossa staining showing crystal deposition in mouse kidneys (n = 3). Representative images and quantitative plots of TUNEL staining to assess tubular epithelial cell apoptosis in mouse kidneys (n = 3). TEM analysis revealed improved damage to renal tubular cells in GSDMD-KO mice compared to WT mice (n = 3). The red arrows indicated the damaged mitochondria. **G, H** Western blot images (**G**) and quantitative plots (**H**) of NLRP3, Caspase-1, GSDMD, IL-1β, and IL-18 expression in kidney tissues from each group of mice (n = 3). **I, J** Western blot images (**I**) and quantitative plots (**J**) of OPN, CD44, and HAS expression in kidney tissues from different groups (n = 3). Data are presented as mean ± SEM. *P < 0.05, **P < 0.01, ***P < 0.001, ns represents non-significant.

To assess the effects and mechanism of GSDMD, we investigated whether GSDMD deficiency influenced Gly-induced renal tubular epithelial cell injury and crystal deposition in mouse models. A mouse model of CaOx nephrocalcinosis was established by intraperitoneal injection of Gly in wild-type mice (WT) or GSDMD-knockout (GSDMD-KO) mice (Fig. 2A). The qRT-PCR and immunohistochemistry confirmed the knockout efficiency of GSDMD (Fig. S2A-S2B). Creatinine (CRE) and blood urea nitrogen (BUN) are common indicators of kidney injury. Although serum CRE and BUN levels were similar in GSDMD-WT and -KO mice in the saline group, GSDMD deficiency significantly mitigated kidney damage induced by Gly (Fig. 2B, C). As a hallmark of inflammasome activation-induced pyroptosis, we assessed the serum levels of IL-18 and IL-1β using ELISA. The concentrations of serum IL-18 and IL-1β were significantly elevated in the stone group of mice compared to the control group, and the lack of GSDMD reversed this trend (Fig. 2D, E). Von Kossa and TUNEL staining revealed that GSDMD deficiency markedly reduced Gly-induced renal crystal deposition and apoptosis of RTECs in the stone model mice (Fig. 2F). Furthermore, western blot assays and immunohistochemistry showed that GSDMD deletion suppressed the expression of NLRP3, Caspase-1, GSDMD, IL-1β, and IL-18 proteins (Fig. 2G, H and S2C).

To further confirm the impact of GSDMD on kidney stone formation, we evaluated the expression of adhesion-related proteins in mouse kidneys from different experimental groups, which play a vital role in the development of kidney stones 43, 44. GSDMD-KO mice exhibited significantly fewer adhesion-related proteins, as shown in the protein levels of osteopontin (OPN), CD44, and hyaluronic acid synthase (HAS), which were notably elevated in the calculous kidney of WT mice (Fig. 2I, J and S2C). TEM results showed that renal tubular cells from the WT + Gly group displayed substantial damage relative to the WT group, as indicated by reduced number of mitochondria, increased autophagosomes, and heightened vacuolation. Moreover, there is a severe disruption in mitochondrial architecture, characterized by mitochondrial swelling and the disorganization, fragmentation, or loss of cristae. Interestingly, GSDMD deficiency partially alleviated the damage to these organelles (Fig. 2F). Together, these findings strongly suggest that GSDMD-mediated pyroptosis promotes tubular cell damage, inflammatory response, and crystal deposition, thereby accelerating kidney stone progression.

### Pharmacological or genetic inhibition of GSDMD inhibited oxalate-induced cell damage and cell-crystal adhesion *in vitro*

**Figure 3:**
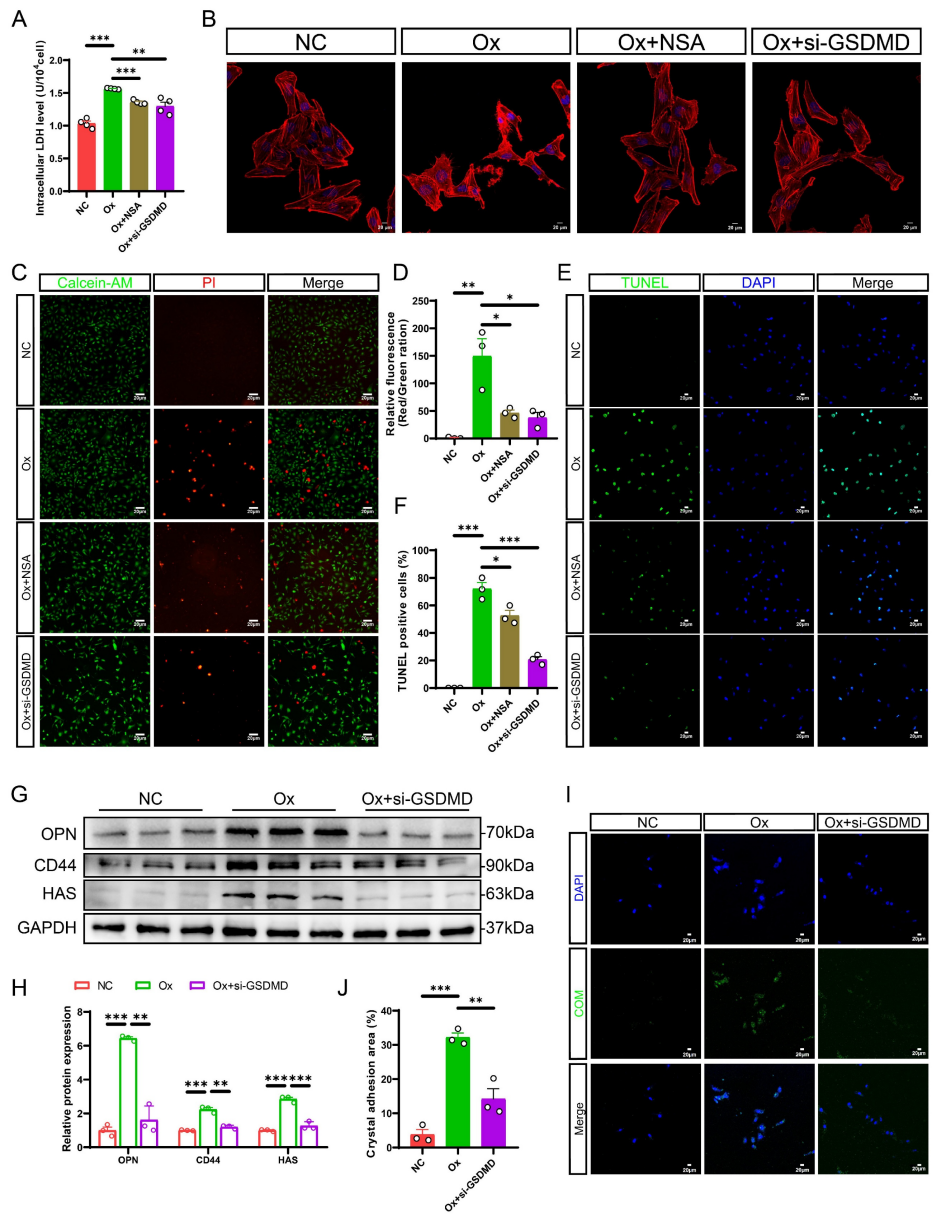
** GSDMD deficiency alleviated Ox-induced cell death and cell-crystal adhesion in HK-2 cells. A** The contents of LDH in the medium of each group of cells (n = 4). **B** Cytoskeletal changes in different treatment groups (n = 3). **C, D** Staining (**C**) and quantitative plots (**D**) of living/dead cells after intervention with Ox in HK-2 cells from different groups (n = 3). **E, F** Representative images (**E**) and quantitative plots (**F**) of TUNEL staining to detect cell apoptosis in HK-2 cells from different groups (n = 3). **G, H** Western blot images (**G**) and quantitative plots (**H**) of OPN, CD44, and HAS expression in HK-2 cells from different groups (n = 3). **I, J** Representative images (**I**) and quantitative plots (**J**) of cell-crystal adhesion of HK-2 cells (n = 3). Data are presented as mean ± SEM. *P < 0.05, **P < 0.01, ***P < 0.001.

To elucidate the critical role of the NLRP3/GSDMD signaling pathway in renal tubular cell pyroptosis *in vitro*, we first transfected small interfering RNA (siRNA) targeting NLRP3 into HK-2 cells. After confirming the knockdown efficiency by measuring the mRNA and protein levels of NLRP3, we constructed an *in vitro* kidney stone model using Ox stimulation (Fig. S3A-S3C). Pretreatment with siRNA alleviated Ox-induced activation of NLRP3 inflammasome and pyroptosis, as evidenced by the downregulation of Caspase-1 and GSDMD-N, and a decrease in IL-1β and IL-18 levels (Fig. S3D-S3E). Next, to investigate whether GSDMD deficiency can alleviate Ox-induced cellular damage, HK-2 cells were treated with a chemical inhibitor, necrosulfonamide (NSA) or *GSDMD* gene silencing siRNA (si-*GSDMD*) to knock down GSDMD expression. GSDMD knockdown efficiency was verified by qRT-PCR and western blot (Fig. S3F-S3H). Lactate dehydrogenase (LDH) release serves as a marker of cell lysis and reflects the level of pyroptosis. Our results indicated that suppressing GSDMD expression significantly reduced LDH release in HK-2 cells after intervention with Ox (Fig. 3A). This finding supported that GSDMD played a crucial role in Ox-induced pyroptosis, which resulted in membrane rupture and the release of intracellular LDH. Moreover, staining of the cellular cytoskeleton revealed substantial damage following Ox exposure, which was markedly mitigated in HK-2 cells treated with si-*GSDMD* or NSA (Fig. 3B). Then, TUNEL staining was utilized to detect Ox-induced cell death, and cell Calcein-AM/PI staining assay was employed to label living cells (green) and dead cells (red), respectively, in order to determine cell viability. As expected, TUNEL and Calcein-AM/PI staining further supported our findings by demonstrating effective inhibition of cell death through siRNA transfection or application of NSA (Fig. 3C-F). All results consistently demonstrate that NLRP3/GSDMD signaling pathway is involved in Ox-induced pyroptosis, and the effect can be prevented by GSDMD deficiency.

Consistent with our *in vivo* data, western blot analysis showed that the protein levels of OPN, CD44, and HAS were increased obviously in the HK-2 cells after intervention with Ox, while these changes were dramatically reversed by *GSDMD* gene silencing (Fig. 3G, H). It has been reported that cell-crystal adhesion is a necessary condition for CaOx calculi formation, and the interaction between RTECs and CaOx crystals plays an essential role in the progression of kidney stones 45. Then, we measured the cell-crystal adhesion status after *GSDMD* knockdown. The cell-crystal adhesion assessments provided evidence that GSDMD deficiency reversed the heightened cell-crystal adhesion capacity observed after intervention with Ox (Fig. 3I, J). In summary, these results strongly suggest that GSDMD-mediated pyroptosis exacerbates tubular cell injury and promotes crystal-cell adhesion, thereby accelerating stone progression.

### NSA treatment mitigated pyroptosis-induced kidney injury and crystal deposition by suppressing the NLRP3/Caspase-1/GSDMD pathway in mice

**Figure 4:**
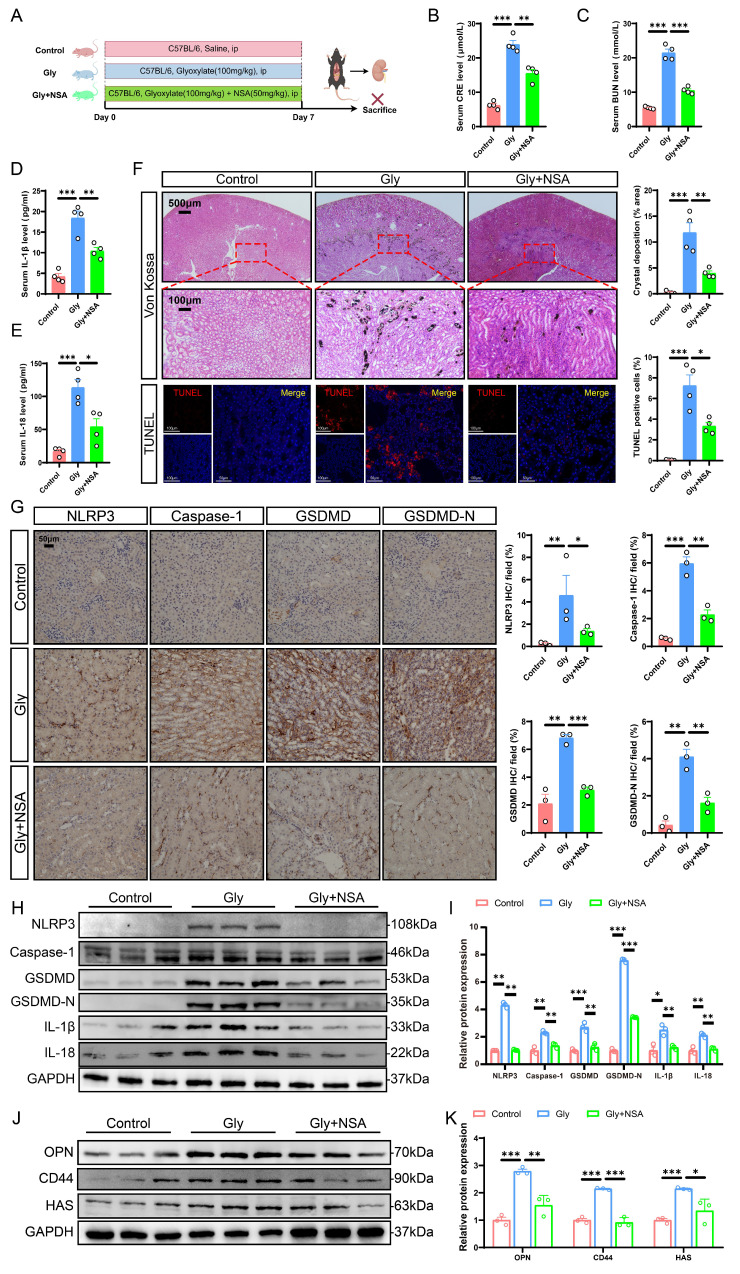
** NSA treatment protected against Gly-induced renal cell pyroptosis and stone aggregation via inhibiting the NLRP3/Caspase-1/GSDMD signaling pathway in the stone model. A** The schematic diagram summarized the animal experiment procedures. ip, intraperitoneal. **B, C** The contents of CRE (**B**) and BUN (**C**) in the serum from different groups (n = 4). **D, E** The contents of IL-1β (**D**) and IL-18 (**E**) in the serum of each group of mice (n = 4). **F** Representative images and quantitative plots of Von Kossa and TUNEL staining showing crystal deposition and cell death in mouse kidneys (n = 4). **G** Representative images for immunohistochemical staining of NLRP3, Caspase-1, GSDMD, and GSDMD-N in kidney tissues from different groups (n = 3). **H, I** Western blot images (**H**) and quantitative plots (**I**) of NLRP3, Caspase-1, GSDMD, GSDMD-N, IL-1β, and IL-18 expression in kidney tissues from different groups (n = 3). **J, K** Western blot images (**J**) and quantitative plots (**K**) of OPN, CD44, and HAS expression in kidney tissues from different groups (n = 3). Data are presented as mean ± SEM. *P < 0.05, **P < 0.01, ***P < 0.001.

NSA is a pyroptosis inhibitor known for its ability to modulate specific pathways associated with cell death and inflammation 46. Our previous studies have reported its inhibitory effects on cell damage and crystal adhesion *in vitro*. To explore the potential role of NSA *in vivo*, we injected the stone mice with either saline or NSA (Fig. 4A). Kidney injury was significantly ameliorated after treatment with NSA, as evidenced by comparable reductions in serum CRE and BUN levels (Fig. 4B, C). Furthermore, NSA treatment decreased serum levels of IL-18 and IL-1β (Fig. 4D, E). Von Kossa staining demonstrated that NSA treatment notably ameliorated CaOx crystal deposition. TUNEL staining showed that Gly increased apoptosis in RTECs, and that NSA treatment significantly reduced the ratio of TUNEL-positive cells (Fig. 4F). Immunohistochemistry results showed that NSA treatment partially reversed the expression of NLRP3, Caspase-1, GSDMD, GSDMD-N, IL-1β, and IL-18 proteins (Fig. 4G and S4). Similar results were demonstrated by western blot (Fig. 4H, I). Importantly, the expression of adhesion-related proteins in the kidneys from the Gly + NSA group was markedly reduced compared with those in the Gly group (Fig. 4J, K and S4). These results support the potential for NSA treatment to ameliorate pyroptosis-induced crystal deposition and renal injury by suppressing the NLRP3/Caspase-1/GSDMD pathway in mice.

### The CHMP4B-mediated cellular repair function was activated during the formation of CaOx nephrolithiasis *in vivo* and *in vitro*

**Figure 5:**
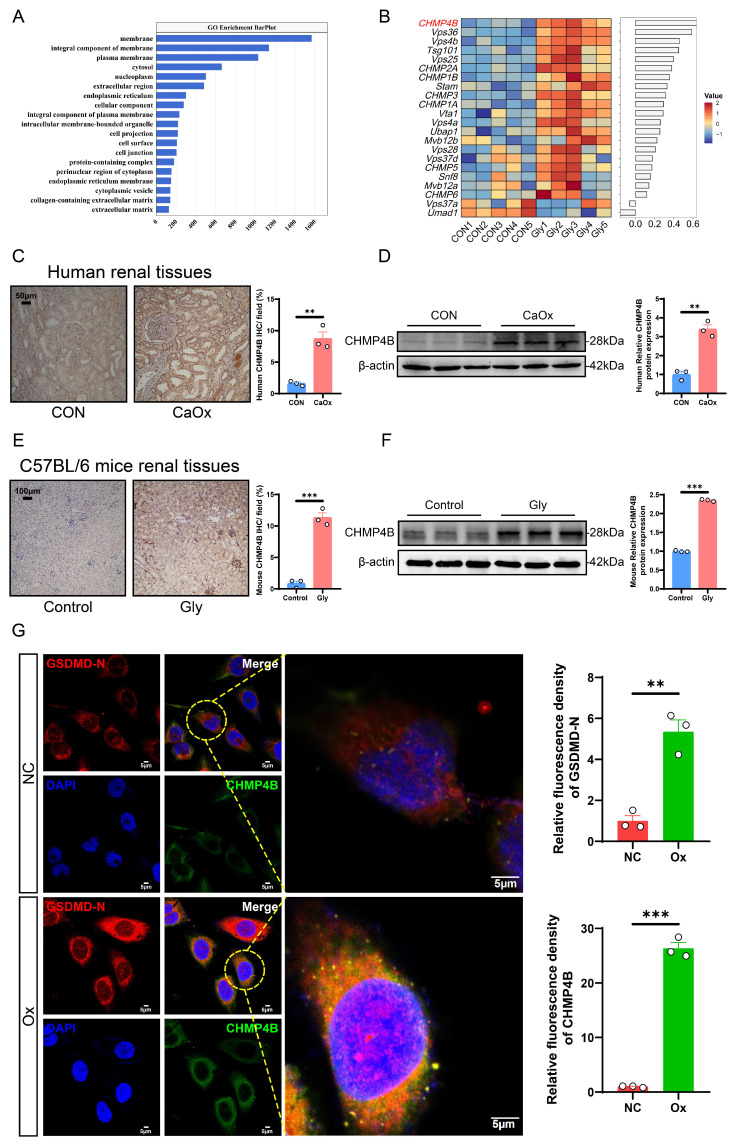
** CHMP4B expression was significantly increased in kidney stone patients, Gly-induced stone mice, and Ox-treated HK-2 cells. A** The GO enrichment analysis is based on transcriptomics data. **B** Heatmap of ESCRT family-related DEGs. **C** Representative images and statistical graphs for immunohistochemical staining of CHMP4B in kidney tissues from normal people and patients with kidney stones (n = 3). **D** Western blot images and quantitative plots of CHMP4B expression in kidney tissues from normal people and patients with kidney stones (n = 3). **E** Representative images and statistical graphs for immunohistochemical staining of CHMP4B in mouse kidneys from the control group and the stone model group (n = 3). **F** Western blot images and quantitative plots of CHMP4B expression in mouse kidneys from the control group and the stone model group (n = 3). **G** Representative images and statistical graphs of colocalization of GSDMD-N (red) and CHMP4B (green) in HK-2 cells after intervention with Ox by immunofluorescence staining (n = 3). Data are presented as mean ± SEM. *P < 0.05, **P < 0.01, ***P < 0.001.

It is well known that cells possess the capability to undergo self-repair following damage. Cellular component analyses revealed that the differentially expressed genes (DEGs) between the control and model groups were mainly enriched in the cell membrane (Fig. 5A). Previous studies reported that ESCRT-dependent membrane repair negatively regulates GSDMD activation and pyroptosis, where it rapidly reseals the damaged cell membrane to restore normal function 27. CHMP4B protein is one of the members of the ESCRT-III complex and plays an important role in plasma membrane repair 47. To further investigate potential cellular damage and repair pathways in the formation of CaOx kidney stones, we focused on a recently described CHMP4B-dependent cell membrane repair mechanism. Analysis of the RNA-seq data revealed that, compared to those observed in the control mice, ESCRT-related genes, including *CHMP4B*, were significantly upregulated in the stone mice (Fig. 5B). Immunohistochemical staining and western blotting of human and mouse kidney tissues further confirmed that CHMP4B expression levels were higher than the controls (Fig. 5C-F). Consistently, western blotting analysis showed that CHMP4B was upregulated in Ox-treated HK-2 cells (Fig. S5A-S5B).

As an integral part of vesicular trafficking, CHMP4B plays important regulatory roles in the transport of diverse signaling molecules. Modifications in CHMP4B localization have the potential to modulate these signaling pathways, thereby affecting cellular reactions to external stimuli 28, 48. We thus probed changes in CHMP4B protein levels and localization by immunofluorescence. In the NC group, CHMP4B was primarily observed to exhibit a uniform distribution throughout the cytoplasm. Notably, upon Ox treatment, the localization of the protein shifted towards the cell membrane, accompanied by an increase in fluorescence intensity, and we found that GSDMD-N colocalized with CHMP4B (Fig. 5G). This observation was similar to the previously reported behavior of CHMP4B during pyroptosis 27. These findings reveal that the cellular repair mediated by CHMP4B is activated during the pathologic process of CaOx kidney stones, which may influence the extent of pyroptosis-induced cellular damage.

### CHMP4B overexpression alleviated pyroptosis-induced cell damage and cell-crystal adhesion most likely by counterbalancing membrane damage in a shedding manner

**Figure 6:**
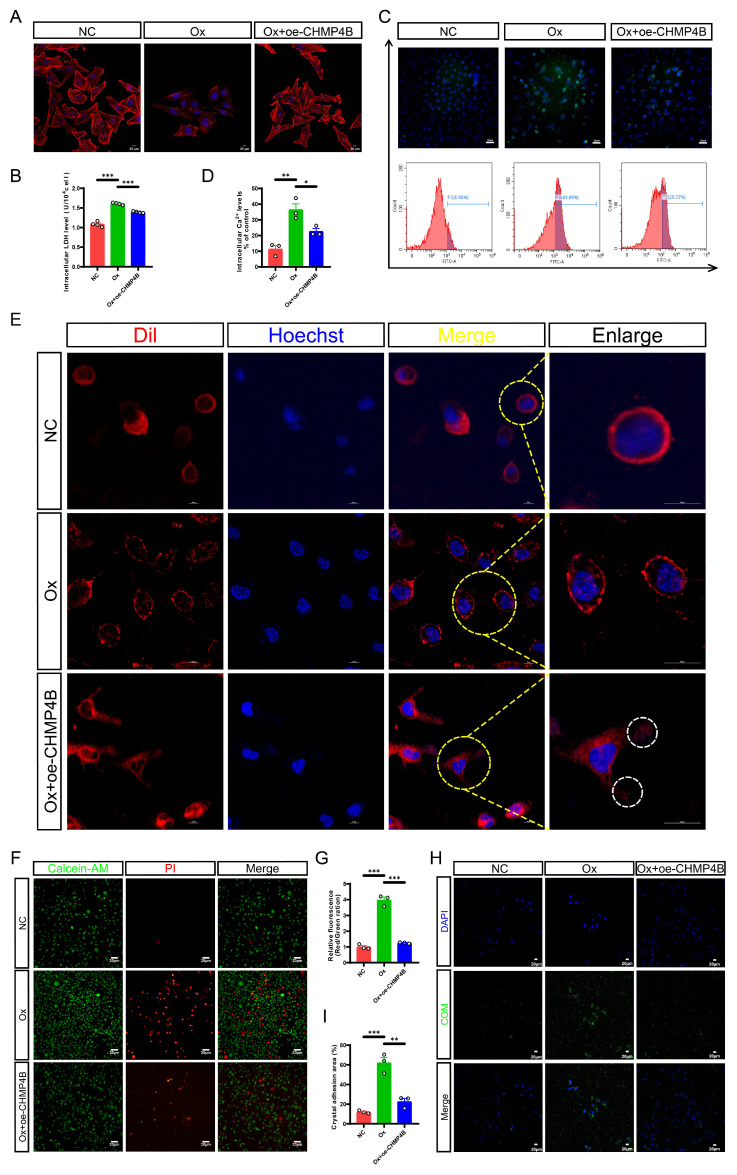
** Enhancing CHMP4B expression partially reversed cell damage, the membrane pore formation, and inhibited cell-crystal adhesion caused by Ox-induced pyroptosis in HK-2 cells. A** Cytoskeletal changes in different treatment groups (n = 3). **B** The contents of LDH in the medium of each group of cells (n = 4). **C, D** The contents of intracellular calcium in HK-2 cells from different groups (n = 3). **E** The structure of the cell membrane changes in different treatment groups (n = 3). Hoechst (blue): nuclei; Dil (red): cellular membrane; White circle: the shedding of cellular membrane. **F, G** Staining (**F**) and quantitative plots (**G**) of living/dead cells after intervention with Ox in HK-2 cells from different groups (n = 3). **H, I** Representative images (**H**) and quantitative plots (**I**) of cell-crystal adhesion of HK-2 cells from different groups (n = 3). Data are presented as mean ± SEM. *P < 0.05, **P < 0.01, ***P < 0.001.

Given the activation of pyroptosis and the upregulation of CHMP4B, we hypothesized that CHMP4B-dependent cell repair was inadequate in counteracting Ox-induced pyroptosis, leading to an imbalance between pyroptosis and cell repair, which ultimately facilitates renal injury and stone formation. To further study the status and molecular mechanism of CHMP4B in renal stone formation, we transfected HK-2 cells with a *CHMP4B* overexpression plasmid to establish cell lines overexpressing CHMP4B (oe-CHMP4B). The qRT-PCR and western blot confirmed the validity of CHMP4B overexpression (Fig. S5C-S5E). Next, we treated normal HK-2 cells and CHMP4B-overexpressing HK-2 cells with Ox for 24 h and subsequently conducted cellular experiments. Results from cytoskeleton staining showed significant damage to the cell cytoskeleton after intervention with Ox. However, this damage was notably reduced in the Ox + oe-CHMP4B group (Fig. 6A). This suggests that CHMP4B may have a protective effect against Ox-induced disruption of the cell cytoskeleton. To further quantify this protective effect, we measured intracellular LDH levels, and the results demonstrated a significant reduction in LDH levels in the Ox + oe-CHMP4B group compared to the Ox group (Fig. 6B). In addition, we knocked down CHMP4B in HK-2 cells, and cell viability was analyzed by CCK-8 assay (Fig. S5F-S5H). We observed that overexpressing CHMP4B exhibited higher cell survival after Ox exposure, while knocking down CHMP4B showed lower cell viability (Fig. S5I-S5J). These results show that CHMP4B has a key role in reversing or repairing Ox-induced cellular injury.

Characteristics of pyroptosis encompass elevated cytoplasmic Ca^2+^ levels, increased cellular volume with rounded morphology, pore formation, and membrane rupture, which are hallmark signs of this process 42, 49. It is widely recognized that CHMP4B is capable of facilitating the repair of the plasma membrane through different mechanisms, including membrane shedding and endocytosis 50.

To assess the potential link between intracellular Ca^2+^ fluctuations and Ox-induced pyroptosis and its subsequent membrane repair, we employed flow cytometry and live-cell confocal imaging to measure and analyze cytosolic Ca^2+^ levels and changes in cellular membrane integrity. Interestingly, flow cytometric analysis revealed that Ox treatment resulted in an elevation of cytosolic Ca^2+^ concentration, whereas overexpression of CHMP4B effectively mitigated the Ox-induced upregulation of Ca^2+^ levels (Fig. 6C, D). We also found that altered membrane architecture in the Ox group, and budding structures were clearly observed in the Ox + oe-CHMP4B group (Fig. 6E). Our study is consistent with the fact that repair mechanisms are activated after the increase in cytosolic Ca^2+^ concentration 51. These results show that the Ca^2+^ fluxes-activated CHMP4B-dependent cell repair plays a promotive role in protecting cells against pyroptosis most likely by membrane shedding.

Then we observed cell death through Calcein-AM/PI staining. It was evident that Ox treatment increased cell death, whereas the overexpression of CHMP4B effectively inhibited cell death (Fig. 6F, G). As expected, the results of cell-crystal adhesion assays indicated that the cell-crystal adhesion increased after Ox intervention, while the overexpression of CHMP4B significantly reduced this change (Fig. 6H, I). Taken together, these data suggested that enhancing the cell repair mediated by CHMP4B could effectively mitigate Ox-induced cell damage and cell-crystal adhesion.

### Overexpression of CHMP4B alleviated kidney injury, stone aggregation, and inflammatory damage caused by pyroptosis in mice

**Figure 7:**
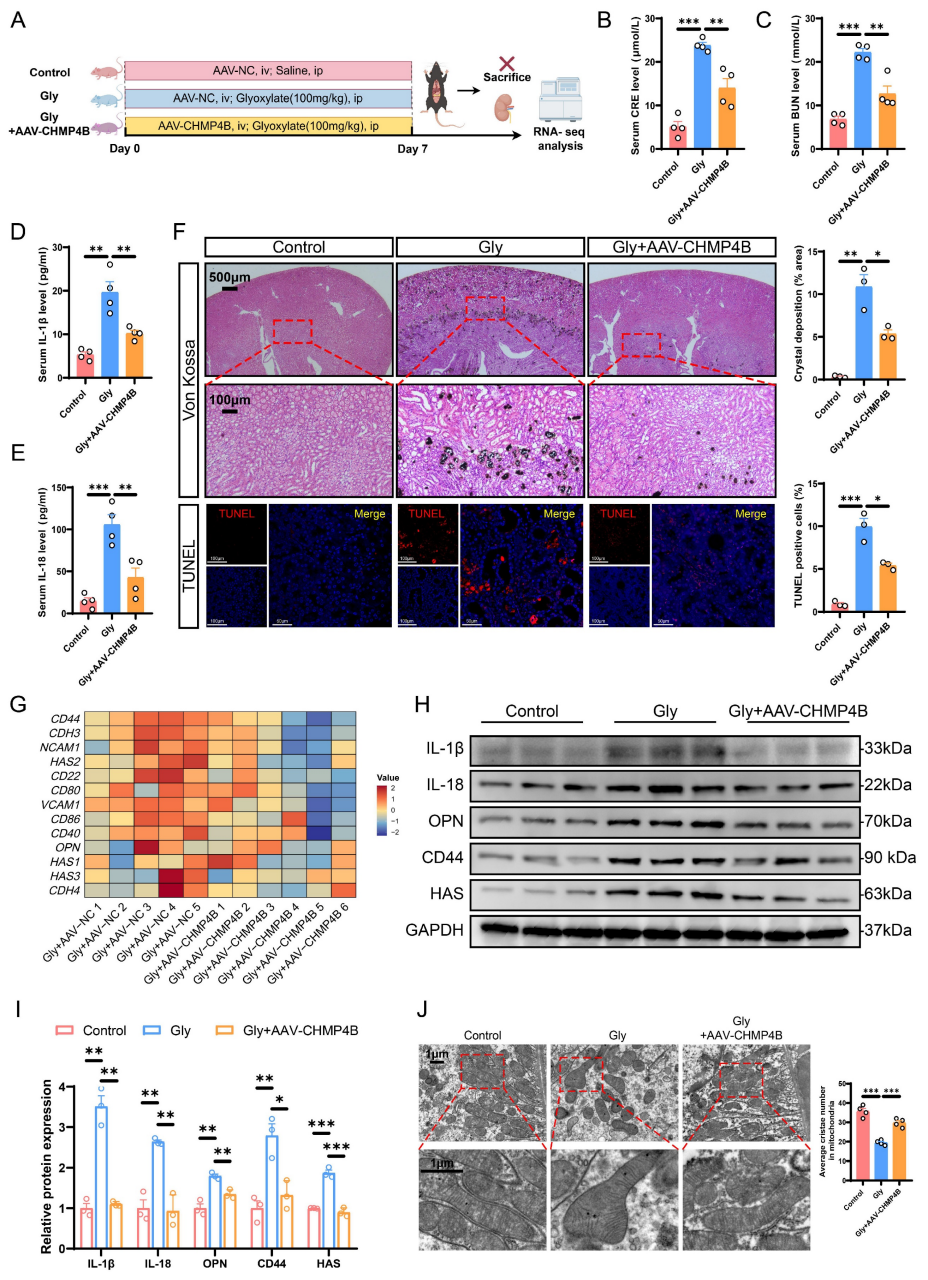
** Overexpression of CHMP4B alleviated kidney injury, stone aggregation and inflammatory damage *in vivo*. A** Experimental schema for establishing renal CHMP4B overexpression mice. Two weeks later, the experimental mice were induced through intraperitoneal injection of Gly (100 mg/kg/d). Kidneys were isolated after 7 days. ip, intraperitoneal; iv, intravenous. **B, C** The contents of CRE (**B**) and BUN (**C**) in the serum of each group of mice (n = 4). **D, E** The contents of IL-1β (**D**) and IL-18 (**E**) in the serum of each group of mice (n = 4). **F** Representative images and quantitative plots of Von Kossa and TUNEL staining showing crystal deposition and cell death in mouse kidneys (n = 3). **G** Heatmap showing the differentially expressed genes that are enriched in cell adhesion molecules (n = 5-6). **H, I** Western blot images (**H**) and quantitative plots (**I**) of IL-1β, IL-18, OPN, CD44, and HAS expression in kidney tissues from each group of mice (n = 3). **J** Representative TEM images showing morphological changes of RTECs from each group of mice (n = 4). Data are presented as mean ± SEM. *P < 0.05, **P < 0.01, ***P < 0.001.

Considering the effect of CHMP4B on promoting cell repair, we investigated whether CHMP4B overexpression could provide protective effects against the formation of CaOx kidney stones *in vivo*. Consequently, we utilized adeno-associated virus (AAV-FLEX-CHMP4B) to induce overexpression of CHMP4B in mouse kidneys. Two weeks after adeno-associated virus injection, CaOx kidney stone mouse models were established (Fig. 7A). Immunohistochemistry confirmed the efficiency of CHMP4B overexpression (Fig. S6A). Levels of CRE and BUN were found to be higher in the Gly group, while significantly lower concentrations were observed following AAV-FLEX-CHMP4B treatment (Fig. 7B, C). Consistently, AAV-FLEX-CHMP4B treatment effectively decreased the IL-18 and IL-1β levels (Fig. 7D, E). Additionally, Von Kossa and TUNEL staining demonstrated that overexpression of CHMP4B reduced stone accumulation and kidney injury (Fig. 7F).

To further investigate the mechanism by which CHMP4B exerts an inhibitory effect on stone formation, RNA-seq was performed in kidneys from Gly-treated AAV-FLEX-NC/CHMP4B-infected mice. The GO enrichment analysis of DEGs indicated that inflammatory response, cell adhesion, and positive regulation of interleukin-1 beta production signaling pathways were significantly downregulated in AAV-FLEX-CHMP4B-treated kidneys (Fig. S6B). As shown by the heatmap of key DEGs, the expression levels of cell damage-related and adhesion-related genes were significantly decreased (Fig. 7G and S6C). Similarly, western blot and immunohistochemical analysis demonstrated that overexpression of CHMP4B decreased levels of adhesion factors and inflammation-related proteins (Fig. 7H, I and S6E). Gene Set Enrichment Analysis (GSEA) indicated that mitochondria-associated genes were significantly enriched (Fig. S6D). Meanwhile, TEM results revealed that overexpression of CHMP4B effectively attenuated the typical reduced numbers of mitochondria and swollen mitochondria with disorganized cristae associated with pyroptosis (Fig. 7J). These results indicate that CHMP4B plays a significant function in the formation of CaOx kidney stone, and that enhancing CHMP4B-mediated membrane repair can mitigate the inflammatory response, cell adhesion, and mitochondrial dysfunction, thereby inhibiting renal injury and stone accumulation.

### Maintaining the balance between GSDMD-mediated pyroptosis and CHMP4B-dependent cell repair protected against CaOx kidney stone formation

The maintenance of cell homeostasis is closely linked to cell health and disease occurrence 52. Cellular structure and function can be severely compromised when cellular damage occurs too frequently or is not adequately repaired. Our previous *in vivo* and *in vitro* studies have shown that inhibition of cell damage alone or enhancement of cell repair can reduce stone production to some extent.

Based on these findings, we further explored the possibility of balancing the relationship between cell damage and repair by combining the reduction of GSDMD-mediated pyroptosis with the enhancement of CHMP4B-dependent cell repair, with the aim of maximizing the restoration of cell homeostasis and significantly inhibiting stone formation. We first conducted *in vitro* experiments to target HK-2 cells by employing the combination of NSA treatment and *CHMP4B* overexpressing plasmid. Subsequently, we observed cell cytoskeleton changes, and the results showed that both NSA treatment and overexpression of CHMP4B attenuated Ox-induced cell damage, with the combination therapy proving to be more effective (Fig. S7A). The results of staining with Calcein-AM/PI and cell-crystal adhesion assays confirmed a similar trend (Fig. S7B-S7C). These findings indicate an enhanced therapeutic efficacy when combining NSA treatment with CHMP4B overexpression therapy under Ox conditions *in vitro*.

**Figure 8:**
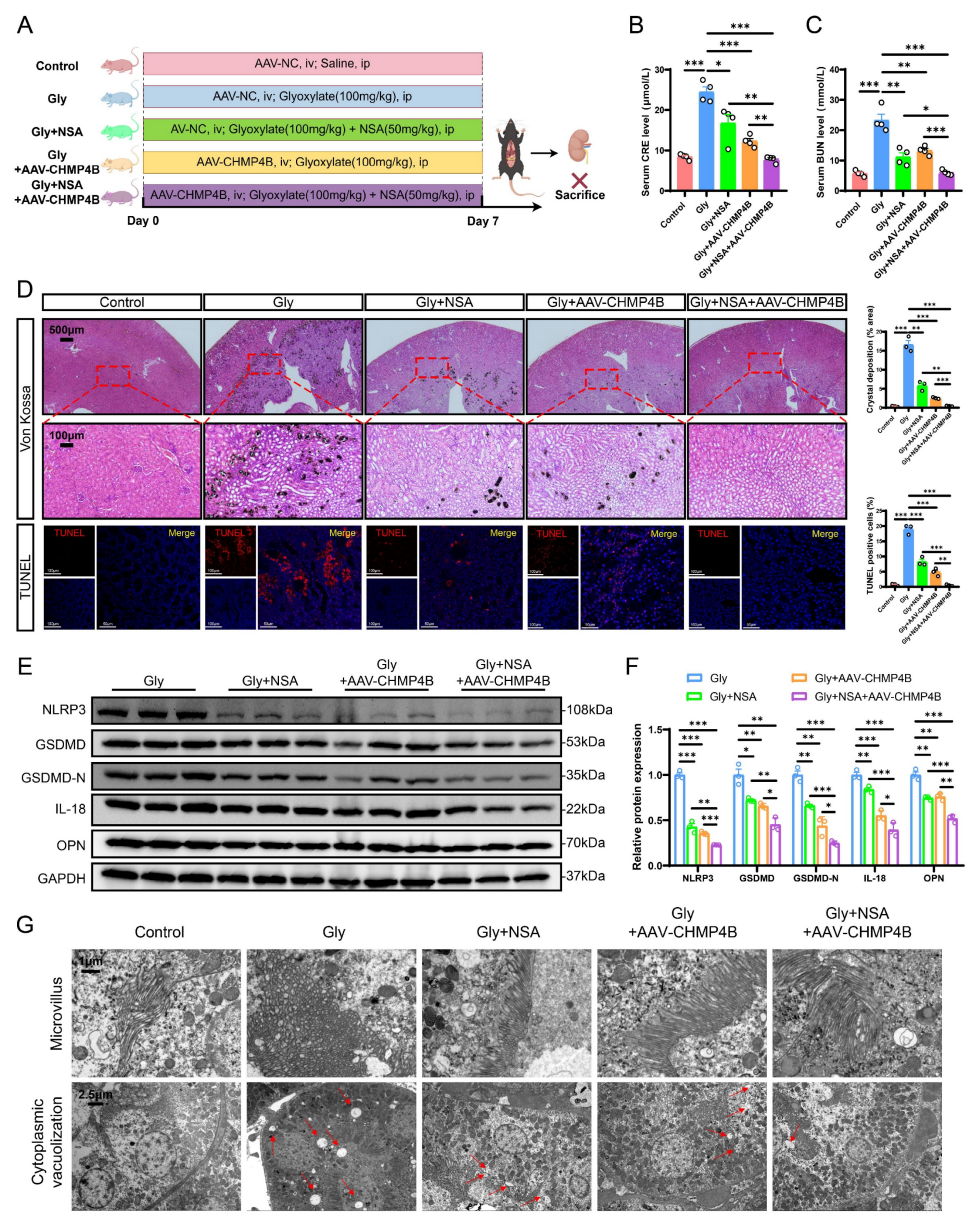
** Inhibition of GSDMD-mediated pyroptosis combined with enhancement of CHMP4B-dependent cell repair could significantly reduce the formation of CaOx kidney stones in mice. A** Experimental schema for establishing a mouse model that enhanced CHMP4B expression while inhibiting GSDMD expression. Two weeks later, the experimental mice were induced through intraperitoneal injection of Gly (100 mg/kg/d). Kidneys were isolated after 7 days. ip, intraperitoneal; iv, intravenous. **B, C** The contents of CRE (**B**) and BUN (**C**) in the serum of each group of mice (n = 4). **D** Representative images and quantitative plots of Von Kossa and TUNEL staining showing crystal deposition and cell death in mouse kidneys (n = 3). **E, F** Western blot images and quantitative plots of NLRP3, GSDMD, GSDMD-N, IL-18, and OPN expression in kidney tissues from each group of mice (n = 3). **G** TEM was used to observe the ultrastructure of microvillus and cytoplasmic vacuolization in renal tissues from different groups (n = 3). The red arrows represented vacuolation. Data are presented as mean ± SEM. *P < 0.05, **P < 0.01, ***P < 0.001.

To determine whether maintaining the GSDMD/CHMP4B balance axis can inhibit stone formation *in vivo*, we administered adeno-associated viruses combined with NSA treatment to the stone group mice (Fig. 8A). NSA treatment reduced CRE and BUN levels (Fig. 8B, C), and attenuated stone aggregation to some extent (Fig. 8D). Moreover, NSA treatment also led to a decrease in the expression of adhesion-related and pyroptosis-associated proteins (Fig. 8E, F). Similarly, CHMP4B overexpression reduced CRE and BUN levels and stone aggregation. (Fig. 8B-D). Very interestingly, the combination of NSA treatment and CHMP4B overexpression significantly mitigated stone aggregation, resulting in a more pronounced inhibitory effect on the serum levels of CRE, BUN, IL-18 and IL-1β, and the expression of adhesion-related and pyroptosis-associated proteins (Fig. 8B-F and S8A-C).

Furthermore, the combinational therapy resulted in lower TUNEL-positive cells (Fig. 8D), and reduced organelle damage as evaluated by TEM (Fig. 8G). Taken together, all these results indicate that the combination of cell repair enhancement with NSA treatment can effectively modulate homeostasis in RTECs, which in turn determines the progression of CaOx kidney stones.

## Discussion

As a global urological disease, the incidence and prevalence of kidney stones are increasing significantly, which brings a heavy burden to families and society 53, 54. In the pathogenesis of renal stone disease, stone formation is often accompanied by a significant increase in inflammation and oxidative free radicals, both of which inevitably lead to cellular structural and functional damage 43. Despite some progress in studying the mechanism of kidney stone formation over recent decades, the specific pathogenesis remains unclear. Recent studies have revealed a close relationship between pyroptosis and kidney stone formation 10-12, 55.

Pyroptosis is a highly inflammatory form of programmed cell death induced by specific pathogens or host immune responses 15. During pyroptosis, Caspase-1 cleaves gasdermin D, generating N-terminal fragments that insert into the plasma membrane, resulting in pore formation, osmotic cell swelling, membrane rupture, and the release of intracellular contents, including pro-inflammatory cytokines and LDH 42, 56. Here, we found that GSDMD-mediated pyroptosis was activated in RTECs from patients with kidney stones and mouse models, and that the expression of pyroptosis-associated proteins was positively correlated with the expression of adhesion molecules in CaOx kidney stones. Our collective results indicate that the NLRP3/GSDMD signaling pathway is involved in the upregulation of inflammatory factors and adhesion molecule-related proteins, and the absence of GSDMD hinders renal inflammation and pyroptosis-induced cell death, thereby attenuating kidney stone formation.

Typically, damaged cells undergo a repair process where they promptly reseal the compromised cell membrane and restore normal function 22. Studies have found that renal tubular epithelial cells facilitate repair following injury by regulating the cell cycle and promoting proliferation 57. Alterations in epigenetic modifications may enhance the expression of genes associated with inflammatory factors, impacting renal repair capacity 58. In addition, the integrity of the cell membrane is essential for cellular survival and functionality. Upon sustaining damage to the cell membrane, cells promptly activate repair mechanisms to address both external stressors and internal injuries 59. This membrane repair mechanism demonstrates varying characteristics across different cell types, particularly in renal cells, where the efficacy of membrane repair directly influences the functional recovery and regenerative capacity of the kidney 60. Increasing evidence suggests that following an acute kidney injury, a synergistic interplay of various repair mechanisms is activated to elicit an adequate reparative response, thereby maintaining renal homeostasis 61-63. However, this repair process may be disrupted in CaOx kidney stones, leading to prolonged cell damage and impaired healing. We pinpointed differential gene expression patterns in kidney tissue from the stone mouse model by genomics data. Pathway enrichment analysis revealed the involvement of several critical pathways, encompassing alterations in cell membrane composition and expression within the ESCRT family. CHMP4B is a member of the ESCRT-III complex, which is responsible for catalyzing the membrane scission event necessary for the formation of intraluminal vesicles (ILVs) within multivesicular bodies (MVBs) 64. This scission event allows for the eventual release of ILVs as exosomes or their fusion with lysosomes for degradation. In addition to its role in MVB formation, CHMP4B has also been implicated in other cellular processes, including cytokinesis (cell division), autophagy (cellular self-degradation), and viral budding 65-67. In the process of cell membrane repair, CHMP4B mediates the multimerization of ESCRT-III to form filamentous, cyclic, or helical structures to repair damaged cell membranes 68. Mutations or dysregulation of CHMP4B have been associated with various diseases, including neurodegenerative disorders like frontotemporal dementia (FTD) and Alzheimer's disease (AD) 69, 70. Our study found that CHMP4B expression levels were elevated in a mouse model of Gly-induced kidney stones but not sufficient to resist GSDMD-mediated pyroptosis. *In vitro*, our studies have shown that upregulation of CHMP4B may attenuate the dysfunction of RTECs during membrane repair, thus inhibiting the process of cellular injury, and alleviating Ox-induced crystal adhesion and inflammatory responses. Mechanistically, our findings are consistent with previous reports that Ca^2+^ fluxes induce activation of the CHMP4B-dependent cell repair machinery, and that CHMP4B can be recruited around the membrane neck to relieve pyroptosis-induced cell damage by removing the membrane pores 27, 51, 71. These findings suggest that strengthening CHMP4B-dependent cellular repair function contributes to the maintenance of cellular homeostasis, which in turn can effectively attenuate cell injury.

Oxidative stress and inflammation are critical factors in the formation of kidney stones, particularly through mechanisms involving lipid peroxidation and cellular stress responses 5, 72. CaOx induces endoplasmic reticulum stress and promotes the production of reactive oxygen species, which can lead to apoptosis and cellular dysfunction 73, 74. Additionally, microRNAs play a vital role as regulators in modulating the response of renal tubular epithelial cells to CaOx-induced injury 11, 75. However, previous studies on the mechanism of kidney stone formation have primarily concentrated on cell damage, and there has been relatively little research focused on the mechanisms of the balance between cellular damage and repair. To investigate the impact of maintaining a balance between GSDMD-mediated cell damage and CHMP4B-dependent cell repair on kidney stone formation, we increased the expression of CHMP4B levels through tail vein injection of adeno-associated virus while reducing pyroptosis by intraperitoneal injection of a GSDMD inhibitor. Furthermore, we examined the combined effect of reducing cell damage and enhancing cell repair on the formation of CaOx kidney stones *in vitro*. Notably, by combining NSA treatment and up-regulation of cellular repair function, we successfully promoted the maintenance of cellular homeostasis, which resulted in a significant reduction in stone aggregation and renal injury. These findings offer novel insights into the mechanisms underlying the balance between damage and repair in renal tubular epithelial cells during renal stone formation. Furthermore, both CHMP4B and GSDMD may be considered as potential therapeutic targets, thereby providing innovative ideas and strategies for the development of future therapeutic interventions.

There are several limitations to our study. We have established that GSDMD-mediated pyroptosis and CHMP4B-dependent cell repair are pivotal in the formation of CaOx kidney stones. CaOx can induce kidney damage and stone deposition by triggering the formation of GSDMD-N mediated membrane pores and the release of IL-18 and IL-1β via activation of the NLRP3 inflammasome. However, the way CHMP4B is expressed and secreted remains uncertain. Further research is needed to understand the molecular mechanisms by which CHMP4B functions under CaOx stimulation. Given the existence of various forms of cell damage in CaOx kidney stone formation, such as apoptosis, necroptosis and ferroptosis, it remains unclear whether CHMP4B-mediated cell repair plays a critical role in other forms of cell damage, and whether other forms of cell repair exist.

**Figure 9:**
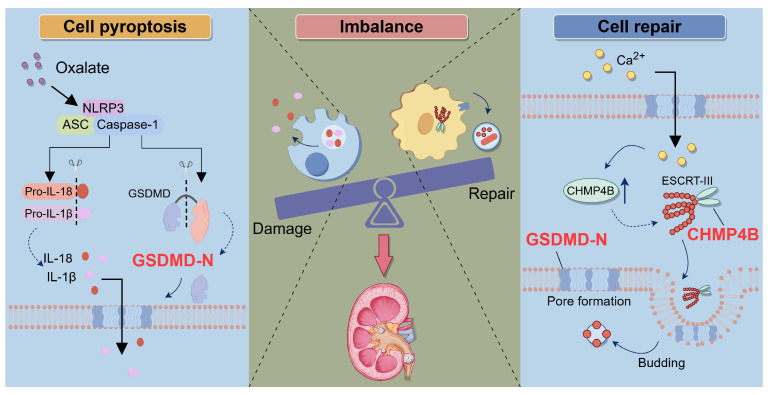
** Schematic illustration of the role of the balance between GSDMD-mediated pyroptosis and CHMP4B-dependent cell repair in CaOx kidney stone disease.** When the kidneys are exposed to exogenous oxalate, the NLRP3 inflammasome is triggered and activated. This activation process further activates Caspase-1, resulting in the cleavage of GSDMD and the generation of GSDMD-N. GSDMD-N induces the formation of membrane pores in the cell membrane of RTECs, which leads to a significant increase in the secretion of IL-1β and IL-18, triggering inflammatory injury. On the other hand, when RTECs undergo pyroptosis, the intracellular concentration of Ca^2+^ increases, which activates the CHMP4B-dependent cellular repair mechanism. CHMP4B concentrates on damaged cellular membrane and repairs these pyroptosis-induced cellular membrane pores by membrane shedding, which inhibits the release of IL-1β and IL-18. However, when Ox-induced pyroptosis exceeds the cellular self-repair capacity, the balance between damage and repair in RTECs will be disrupted. This leads to exacerbated damage in RETCs and may further promote the release of IL-1β and IL-18, as well as upregulation of crystal adhesion proteins. This persistent inflammatory microenvironment further exacerbates damage to RTECs, ultimately leading to the formation of kidney stones.

In conclusion, our study revealed that the imbalance between GSDMD-mediated cell damage and CHMP4B-dependent cell repair exacerbated the formation of CaOx kidney stones, offering new insights into the pathogenesis of kidney stone disease (Fig. 9). By elucidating the signaling pathway of tubular epithelial cell damage and repair processes, we can discover new interventions to sustain the balance between damage and repair, thereby providing novel and more effective strategies for preventing and treating kidney stone disease.

## Supplementary Material

Supplementary figures and tables.

## Abbreviations

RTECs: renal tubular epithelial cells
GSDMD: gasdermin D
CaOx: calcium oxalate
NLRP3: NOD-like receptor protein 3
CHMP4B: charged multivesicular body protein 4B
Gly: glyoxylate
GSDMD-N: N-terminal fragment of GSDMD
DAMPs: damage-associated molecular patterns
ESCRT: endosomal sorting complex required for transport
NSA: necrosulfonamide
AAV: adeno-associated virus
IL-1β: interleukin-1β
IL-18: interleukin-18
qRT-PCR: quantitative real-time PCR
CD44: cluster of differentiation 44
CRE: creatinine
BUN: blood urea nitrogen
OPN: osteopontin
HAS: hyaluronic acid synthase
TEM: transmission electron microscopy
Ox: oxalate
LDH: lactate dehydrogenase
DEGs: differentially expressed genes
GSEA: Gene Set Enrichment Analysis
ILVs: intraluminal vesicles
MVBs: multivesicular bodies
FTD: frontotemporal dementia
AD: Alzheimer's disease
