# Supplementary material for: Regulating the balance between GSDMD-mediated pyroptosis and CHMP4B-dependent cell repair attenuates calcium oxalate kidney stone formation
Source: Int J Biol Sci. 2025 Apr 22;21(7):3099–121. doi: 10.7150/ijbs.105415 (PMC12080391; doi:10.7150/ijbs.105415)
Supplement: Supplementary file 1 — Supplementary figures and tables. [file ijbsv21p3099s1.pdf]

Supplementary Materials for

Regulating the balance between GSDMD-mediated pyroptosis and CHMP4B-dependent cell repair attenuates calcium oxalate kidney stone formation

Shushuai Yang[1*], Yuanjiong Qi[1*], Yue Chen[1*], Hailong Kong[1], Bin Han[1], Zhongsheng Peng[1], Chenglong Xu[1], Bohan Wang[2#], Liqun Chen[3#], Shiyong Qi[1#]

1. Department of Urology, Tianjin Institute of Urology, The Second Hospital of Tianjin Medical University, Tianjin 300211, China
2. Department of Urology, The Second Affiliated Hospital, School of Medicine, Zhejiang University, Hangzhou 310000, China
3. Medical College, Academy of Medical Engineering and Translational Medicine, Tianjin University, Tianjin 300072, China

[*]These authors contributed equally to this work.

[#]Corresponding author: Bohan Wang, E-mail: wangbohan@zju.edu.cn; Liqun Chen, E-mail: chenliqunlab@163.com; Shiyong Qi, E-mail: yongshiqi_qsy@tmu.edu.cn.

This file includes:

1. Supplementary figures and relative supplementary figure legends (Figure S1 to S8)
2. Supplementary Tables (Table S1 to S3)

Supplementary figures

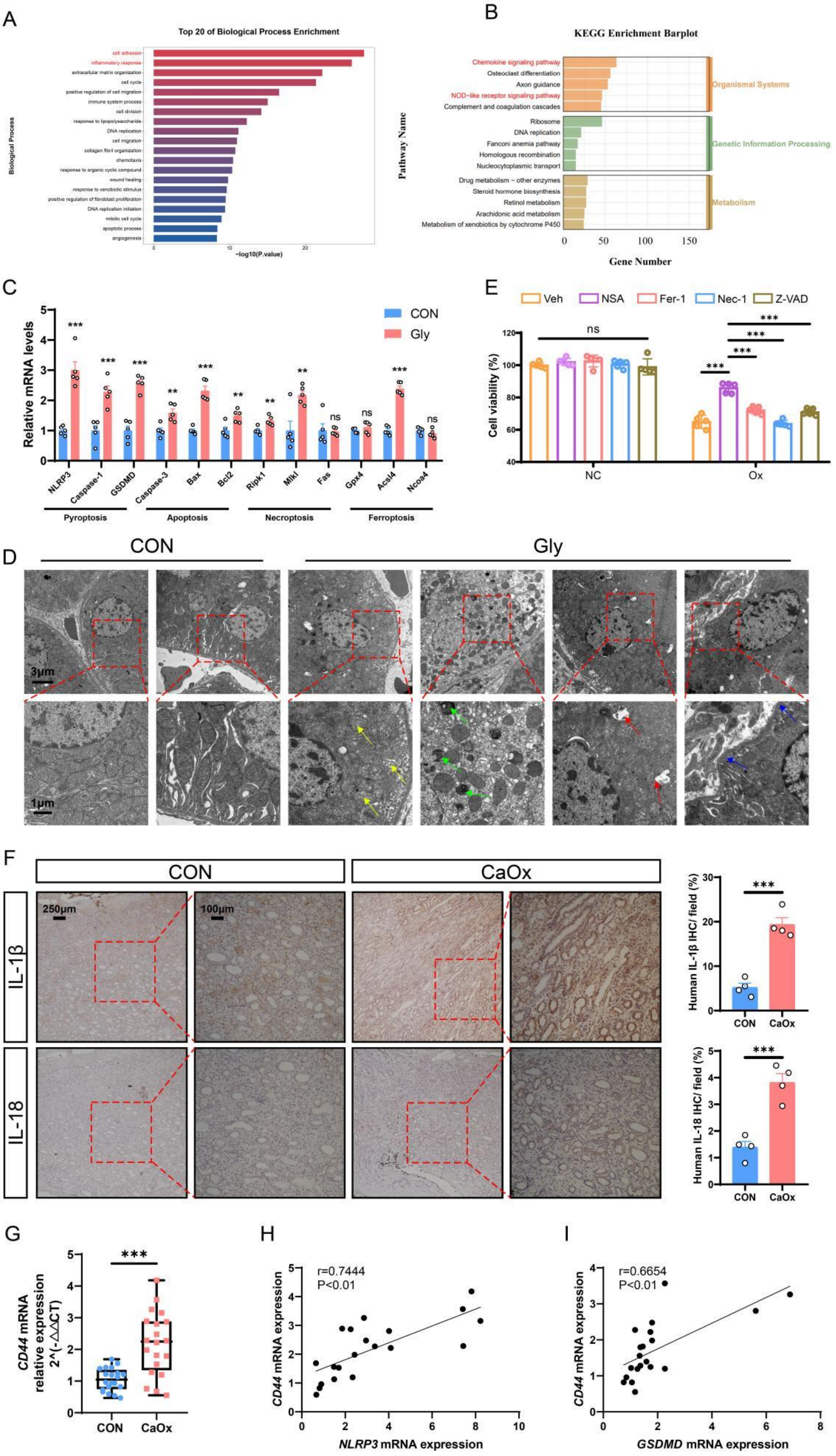

Figure S1. Pyroptosis and inflammation serve as potential factors for the development of renal stones in both patients and mice. A Gene ontology (GO) enrichment analysis of differentially expressed genes. **B** Kyoto Encyclopedia of Genes and Genomes (KEGG) enrichment analysis of differentially expressed genes. **C** Relative mRNA expression of genes related with pyroptosis (*NLRP3*, *Caspase-1*, and *GSDMD*), apoptosis (*Caspase-3*, *Bax*, and *Bcl2*), necroptosis (*Ripk1*, *Mlkl*, and *Fas*), and ferroptosis (*Gpx4*, *Acsl4*, and *Ncoa4*) in mouse kidney tissues from the RNA-Seq data. **D** The ultrastructure of renal tubular epithelial cells in the control and stone groups was observed by TEM (n = 3). The yellow arrows indicate swollen mitochondria. The green arrows indicate autophagy vacuoles. The red arrows indicate cytoplasmic vacuolisation. The blue arrows indicated membrane protrusion. **E** The viability of HK-2 cells in different groups was measured by CCK8 assay (n = 5). **F** Representative images and statistical graphs for immunohistochemical staining of IL-1β and IL-18 in kidney tissues from normal people and patients with kidney stones (n = 4). **G** Relative mRNA expression of *CD44* was assessed by qRT-PCR in 20 normal people and 20 patients with kidney stones (n = 20). **H** The linear regression analysis of the relevance between *NLRP3* mRNA expression and *CD44* mRNA expression ($r = 0.7444$, $P < 0.01$, $n = 20$). **I** The linear regression analysis of the relevance between *GSDMD* mRNA expression and *CD44* mRNA expression ($r = 0.6654$, $P < 0.01$, $n = 20$). Data are presented as mean ± SEM. $^{**}P < 0.01$, $^{***}P < 0.001$, ns represents non-significant.

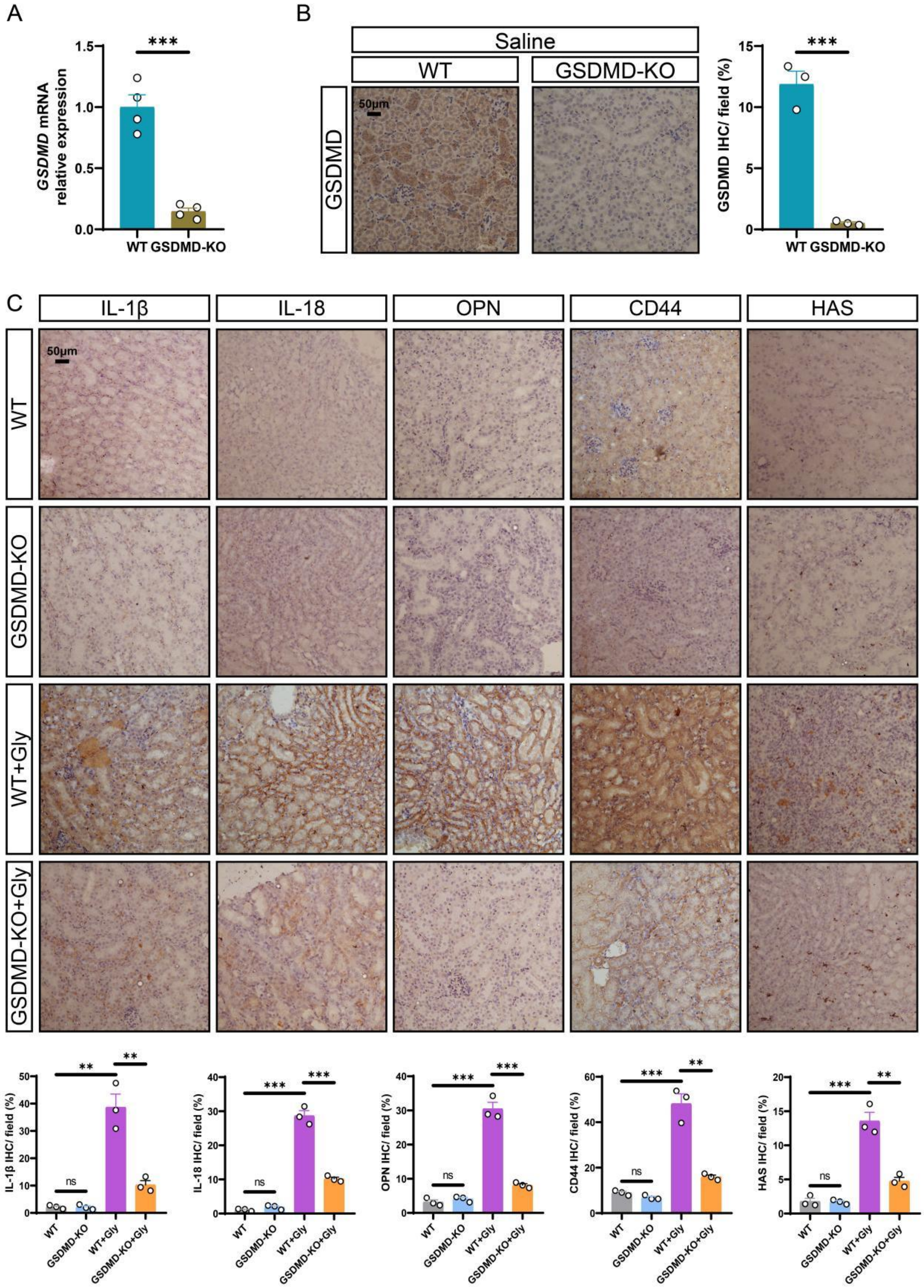

Figure S2. GSDMD deficiency significantly alleviated renal inflammation and crystal adhesion in the stone model. A Relative mRNA expression of *GSDMD* was assessed by qRT-PCR in different kidney tissues (n = 4). **B** The knockout efficiency of GSDMD was

confirmed by immunohistochemistry (n = 3). **C** Representative images and statistical graphs for immunohistochemical staining of IL-1β, IL-18, OPN, CD44, and HAS in kidney tissues from different groups (n = 3). Data are presented as mean ± SEM. $^{**}P < 0.01$, $^{***}P < 0.001$, ns represents non-significant.

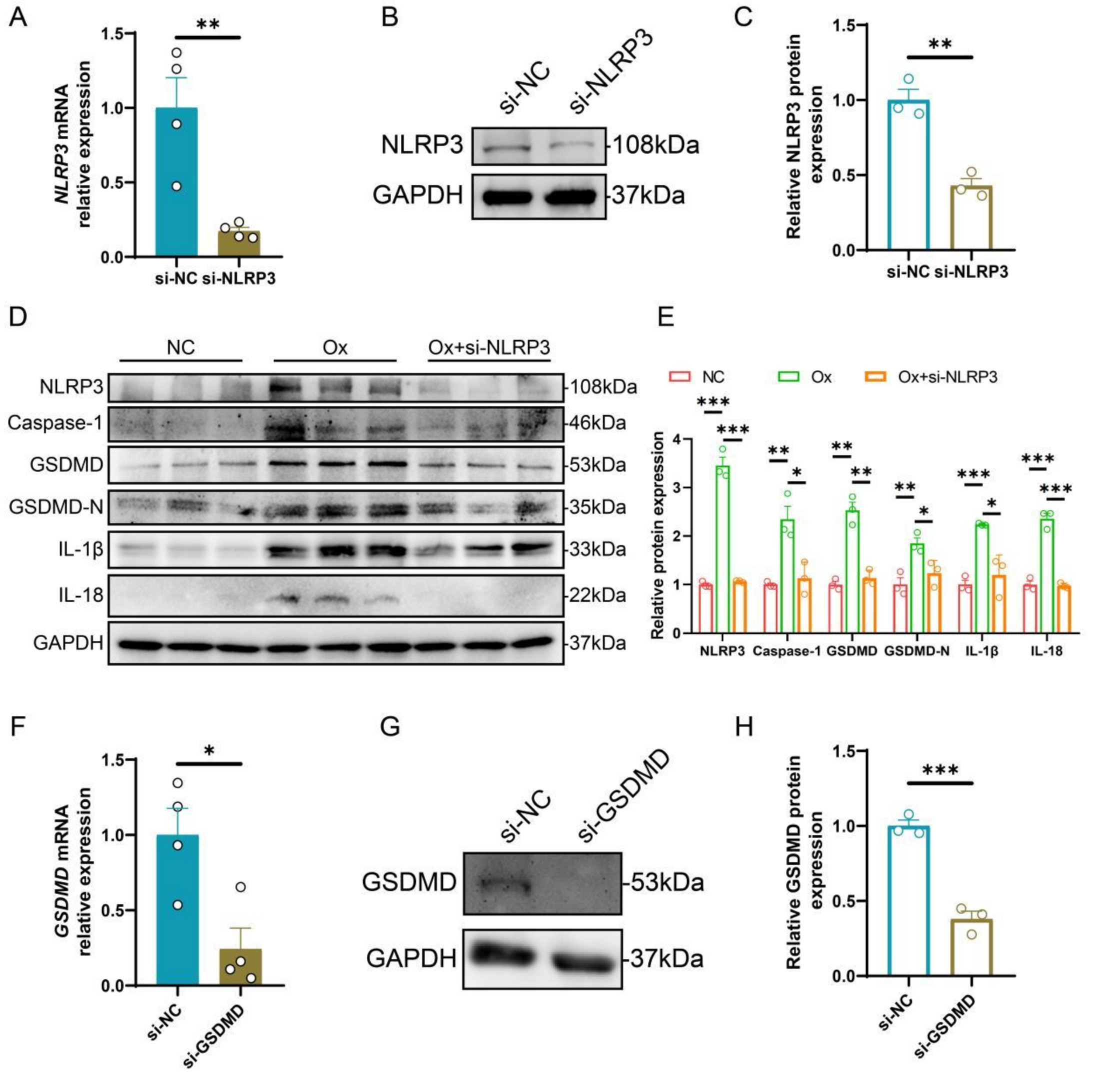

Figure S3. GSDMD-mediated pyroptosis was caused by NLRP3 signaling pathway in HK-2 cells. A-C HK-2 cells were transfected with the si-*NC* or si-*NLRP3* for 48 h, the mRNA level of *NLRP3* was detected by qRT-PCR (n = 4), and the protein level of NLRP3 was detected by Western blotting (n = 3). **D, E** Western blot images (**D**) and quantitative plots

(**E**) of NLRP3, Casepase-1, GSDMD, GSDMD-N, IL-1β, and IL-18 expression in HK-2 cells from different groups (n = 3). **F-H** HK-2 cells were transfected with the si-*NC* or si-*GSDMD* for 48 h, the mRNA level of *GSDMD* was detected by qRT-PCR (n = 4), and the protein level of GSDMD was detected by Western blotting (n = 3). Data are presented as mean ± SEM. **P* < 0.05, ***P* < 0.01, ****P* < 0.001.

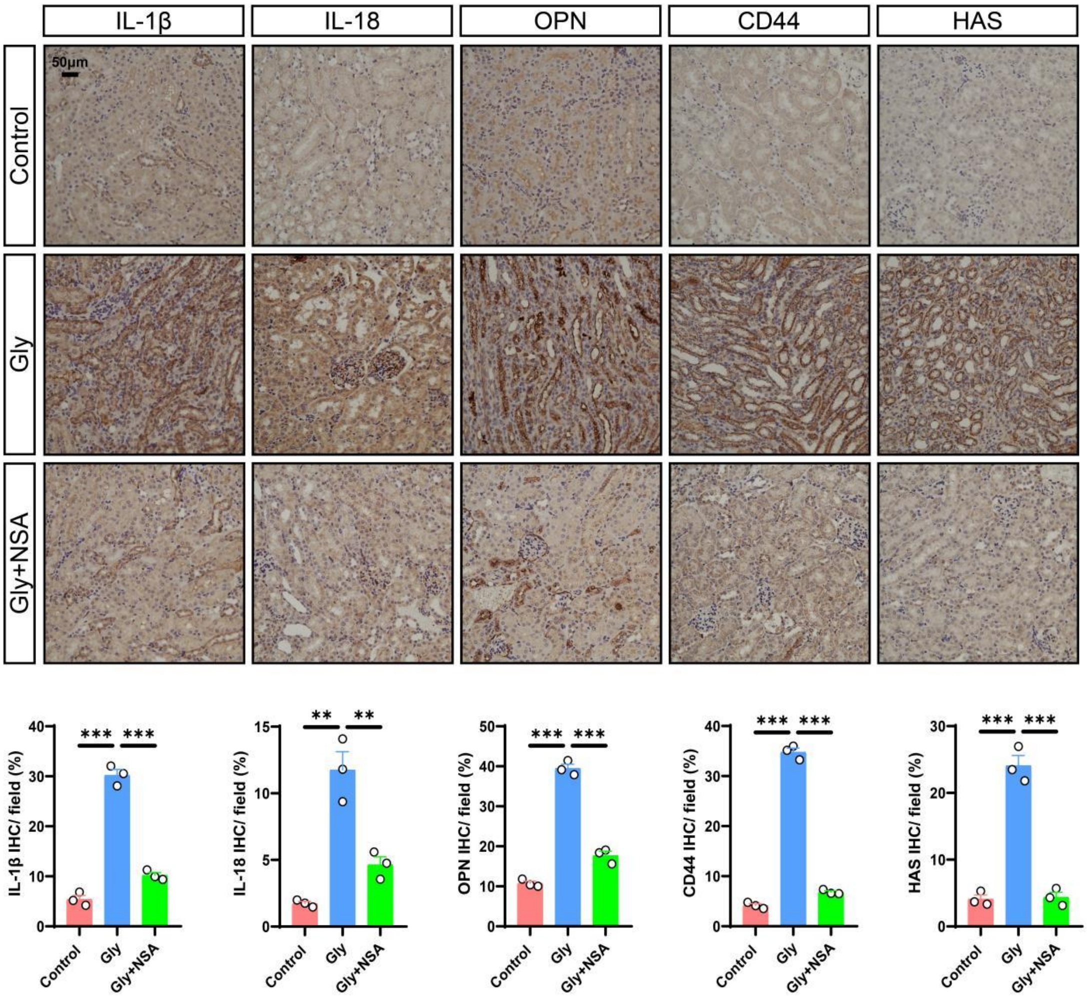

Figure S4. NSA treatment significantly alleviated renal inflammation and crystal adhesion in the stone model. Representative images and statistical graphs for

immunohistochemical staining of IL-1β, IL-18, OPN, CD44, and HAS in kidney tissues from different groups (n = 3). Data are presented as mean ± SEM. **$P < 0.01$, ***$P < 0.001$.

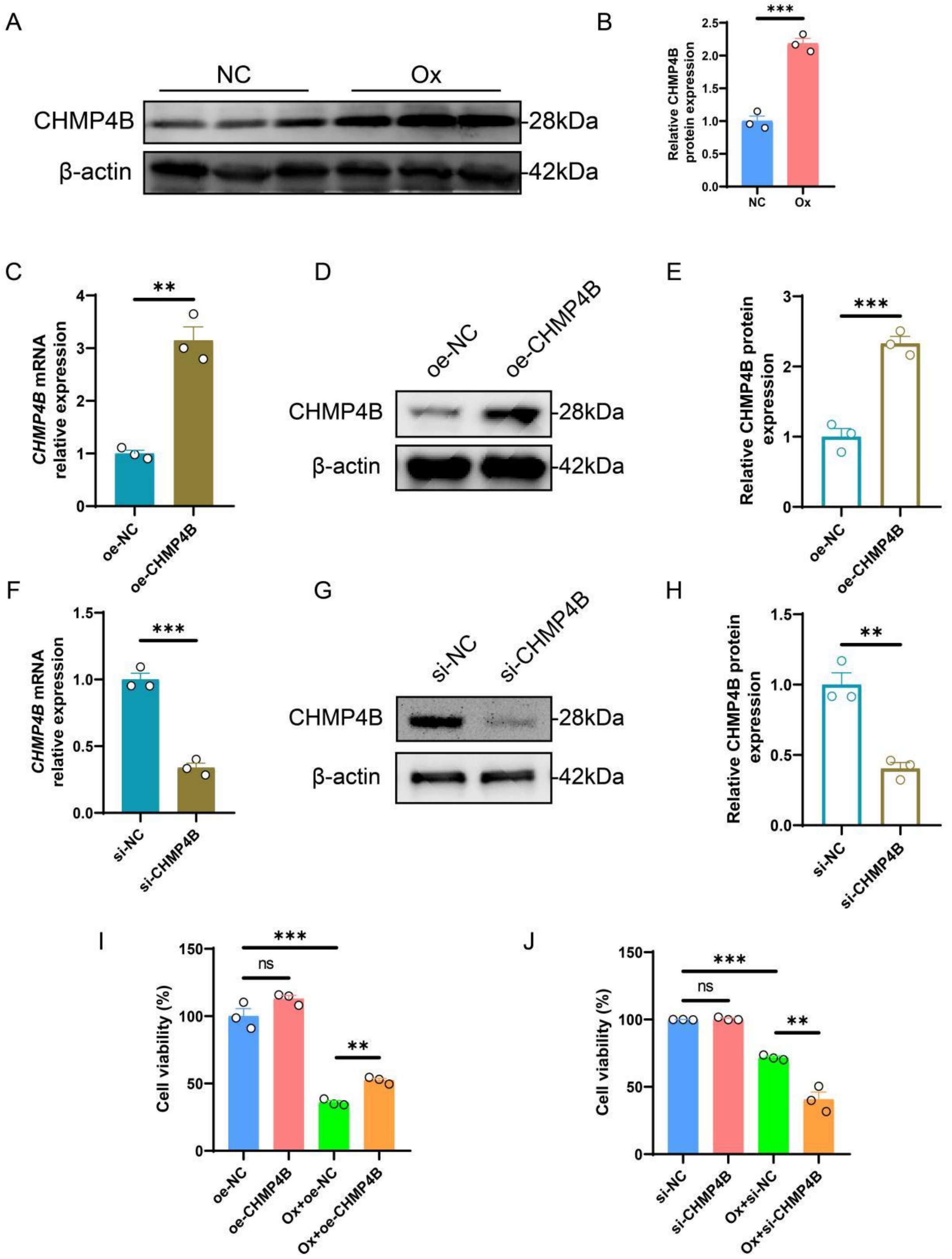

Figure S5. CHMP4B expression was significantly increased in Ox-treated HK-2 cells. A, B Western blot images (**A**) and quantitative plots (**B**) of CHMP4B expression in HK-2 cells after intervention with Ox (n = 3). **C-E** HK-2 cells were transfected with the oe-*NC* or oe-*CHMP4B* for 48 h, the mRNA level of *CHMP4B* was detected by qRT-PCR, and the protein level of CHMP4B was detected by Western blotting (n = 3). **F-H** HK-2 cells were transfected with the si-*NC* or si-*CHMP4B* for 48 h, the mRNA level of *CHMP4B* was detected by qRT-PCR, and the protein level of CHMP4B was detected by Western blotting (n = 3). **I, J** The viability of HK-2 cells in different groups was evaluated by a CCK8 assay (n = 3). Data are presented as mean ± SEM. $^{**}P < 0.01$, $^{***}P < 0.001$, ns represents non-significant.

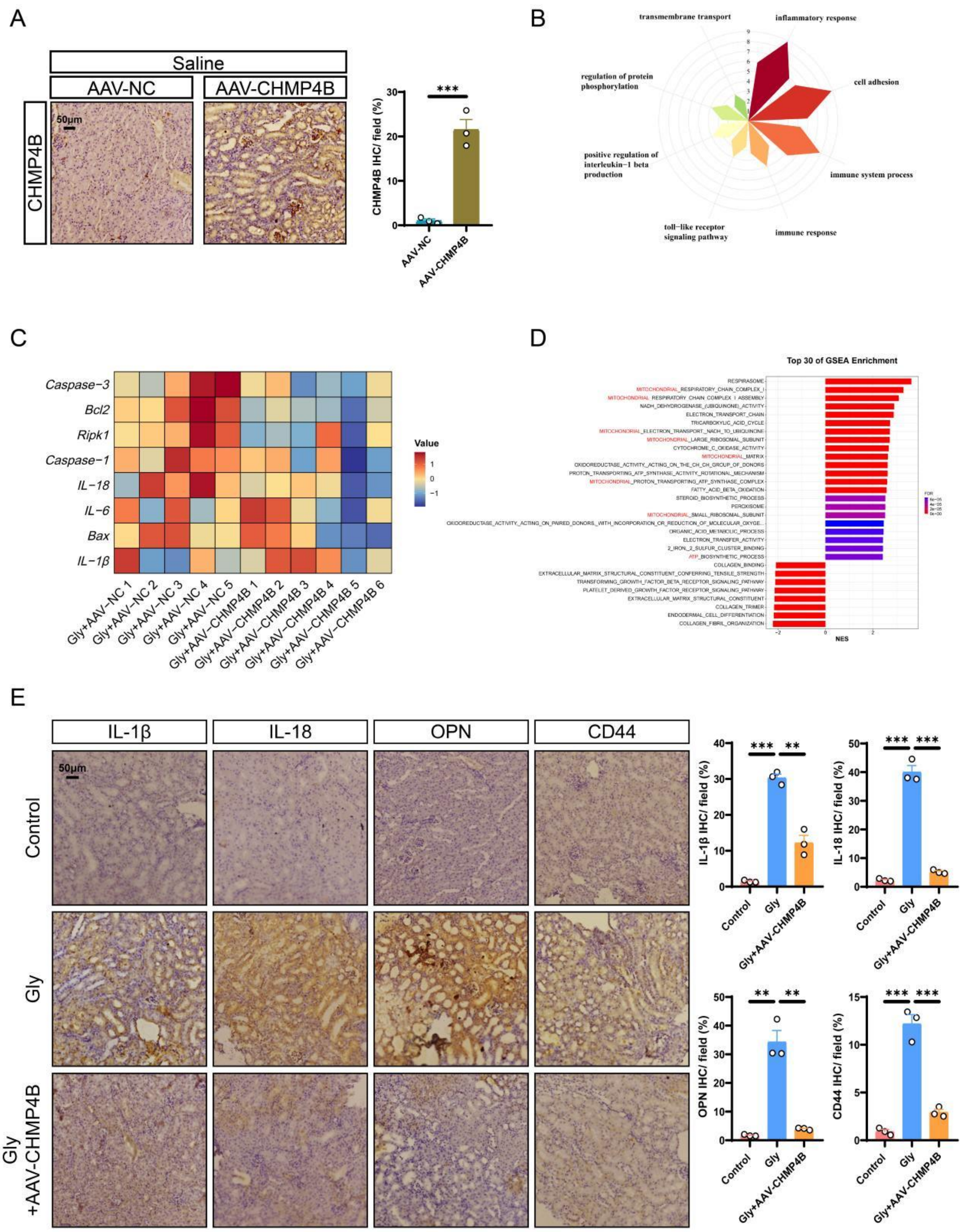

Figure S6. CHMP4B overexpression partially reversed the Gly-induced renal tubular epithelial cell injury *in vivo*. **A** The efficiency of CHMP4B overexpression was confirmed by immunohistochemistry (n = 3). **B** GO enrichment analysis of differentially expressed genes. **C** Heatmap showing the differentially expressed genes that are enriched in cell

damage-related genes (n = 5-6). **D** Gene Set Enrichment Analysis (GSEA) of differentially expressed genes. **E** Representative images and statistical graphs for immunohistochemical staining of IL-1β, IL-18, OPN, and CD44 in kidney tissues from different groups (n = 3). Data are presented as mean ± SEM. $^{**}P < 0.01$, $^{***}P < 0.001$.

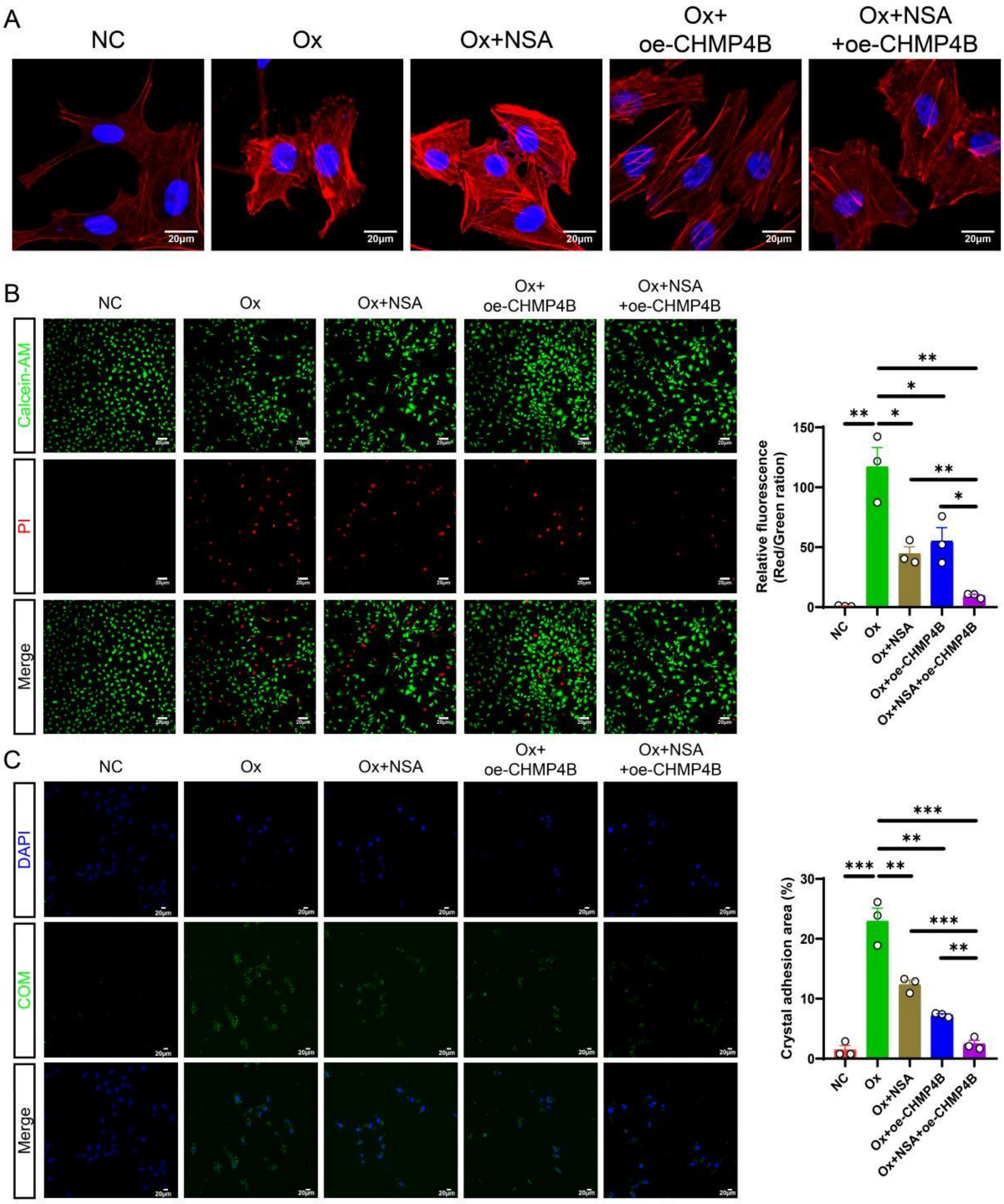

Figure S7. Combination NSA treatment with CHMP4B gene amplification displayed better inhibition of cell death and cell-crystal adhesion caused by Ox *in vitro*. A

Cytoskeletal changes in HK-2 cells from different treatment groups (n = 3). **B** Calcein AM/PI staining and quantitative plots of living/dead cells after intervention with Ox in HK-2 cells from different groups (n = 3). **C** Representative images and quantitative plots of cell-crystal adhesion of HK-2 cells from different treatment groups (n = 3). Data are presented as mean ± SEM. $*P < 0.05$, $**P < 0.01$, $***P < 0.001$.

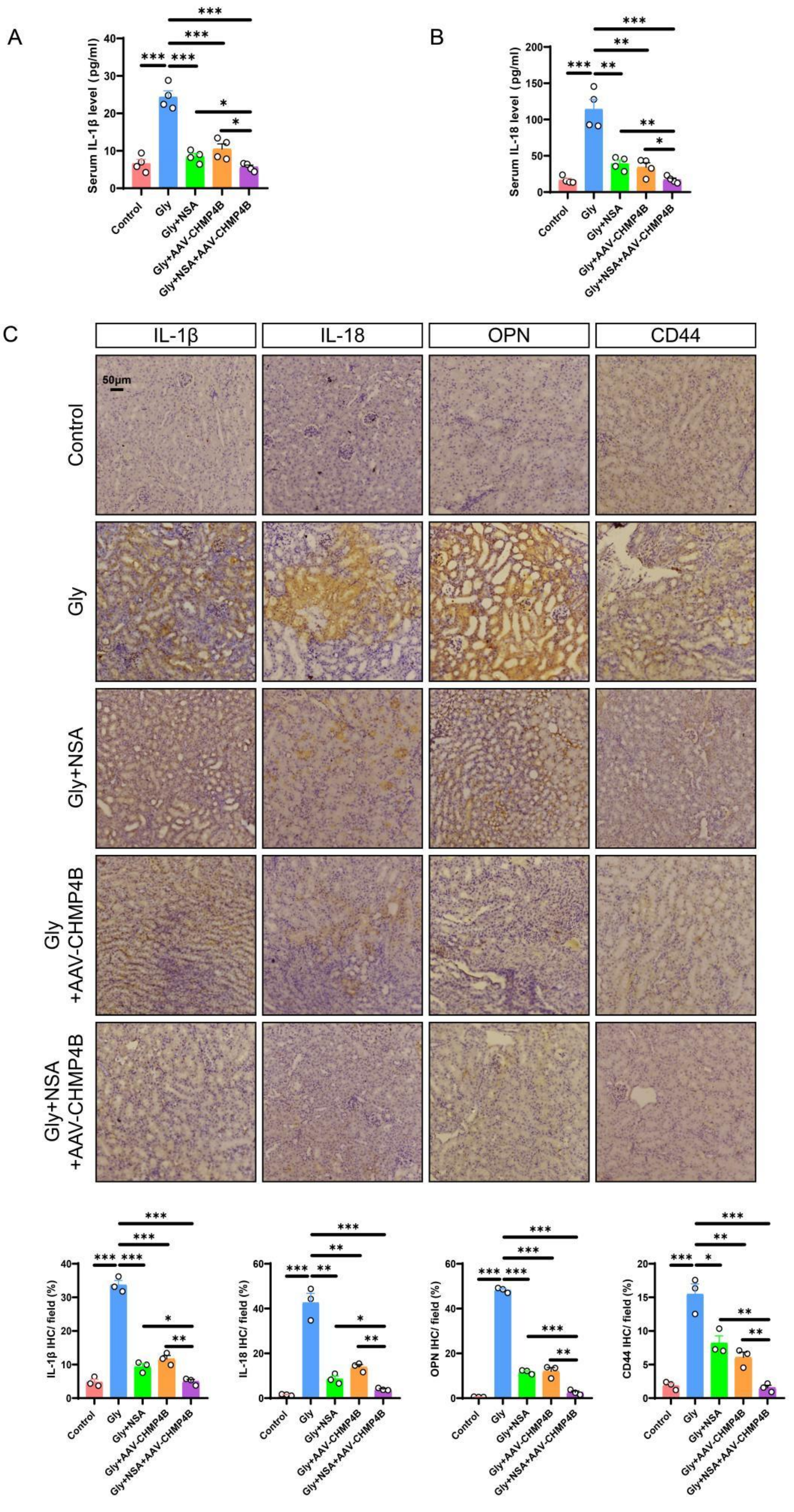
A
Serum IL-1β level (pg/ml)
B
Serum IL-18 level (pg/ml)
Control
Gly
Gly+NSA
Gly+AAV-CHMP4B
Gly+NSA+AAV-CHMP4B
C
IL-1β
IL-18
OPN
CD44
50μm
Control
Gly
Gly+NSA
Gly +AAV-CHMP4B
Gly+NSA +AAV-CHMP4B
IL-1β IHC/ field (%)
IL-18 IHC/ field (%)
OPN IHC/ field (%)
CD44 IHC/ field (%)

Figure S8. The combination of NSA treatment and CHMP4B overexpression could protect against Gly-induced renal tubular epithelial cell injury *in vivo*. A, B The contents of IL-1β (**A**) and IL-18 (**B**) in the serum of each group of mice (n = 4). **C** Representative images and statistical graphs for immunohistochemical staining of IL-1β, IL-18, OPN, and CD44 in kidney tissues from different groups (n = 3). Data are presented as mean ± SEM. $^{*}P < 0.05$, $^{**}P < 0.01$, $^{***}P < 0.001$.

Supplementary Tables

Table S1. Clinical characteristics of normal individuals and kidney stone patients.

Normal individuals

No.	Gender	Age
1	F	65
2	F	68
3	F	61
4	M	68
5	M	54
6	F	68
7	M	66
8	M	68
9	M	43
10	M	50

11	F	62
12	M	68
13	M	43
14	M	51
15	F	57
16	F	59
17	F	32
18	M	42
19	M	43
20	M	51

Kidney stone patients

No.	Gender	Age	Side	Location	CT value
1	F	54	R	Kidney	1004
2	M	79	L	Ureteral	1213
3	M	68	R	Ureteral	1038
4	M	42	R	Ureteral	1023
5	M	36	L	Kidney	1221
6	M	36	L	Kidney	1284
7	F	32	L	Kidney	1393

8	M	60	L	Kidney	1605
9	F	59	L	Ureteral	1156
10	M	40	L	Kidney	1110
11	M	50	L	Kidney	1257
12	M	33	L	Kidney	1596
13	F	57	L	Kidney	1337
14	M	59	R	Kidney, Ureteral	1667
15	M	36	L	Kidney	1511
16	M	26	L	Ureteral	913
17	M	48	L	Kidney	1035
18	M	55	L	Kidney	1275
19	M	53	R	Kidney	1376
20	F	54	L	Kidney	1282

F = Female; M = Male; R = Right; L = Left.

Table S2. Primary and secondary antibodies used in western blot and immunohistochemical analysis.

Antibody	Catalog Number	Manufacturer	Origin
NLRP3	68102-1-Ig	Proteintech	Wuhan, China

Caspase-1	22915-1-AP	Proteintech	Wuhan, China
GSDMD	20770-1-AP	Proteintech	Wuhan, China
GSDMD-N	#DF13758	Affinity Biosciences	Jiangsu, China
CHMP4B	13683-1-AP	Proteintech	Wuhan, China
IL-1β	26048-1-AP	Proteintech	Wuhan, China
IL-18	10663-1-AP	Proteintech	Wuhan, China
OPN	22952-1-AP	Proteintech	Wuhan, China
CD44	60224-1-Ig	Proteintech	Wuhan, China
HAS	15609-1-AP	Proteintech	Wuhan, China
β-actin	81115-1-RR	Proteintech	Wuhan, China
GAPDH	60004-1-Ig	Proteintech	Wuhan, China
HRP, Goat Anti-Rabbit IgG	A21020	Abbkine	Wuhan, China
HRP, Goat Anti-Mouse IgG	A21010	Abbkine	Wuhan, China

Table S3. Primer sequences for qRT-PCR.

Primer name	Forward primer	Reverse primer

NLRP3 (Human)	5′-CGTGAGTCCCATTAAGATGGAGT-3′	5′-CCCGACAGTGGATATAGAACAGA-3′
GSDMD (Human)	5′-GAGTGTGGCCTAGAGCTGG-3′	5′-GGCTCAGTCCTGATAGCAGTG-3′
CHMP4B (Human)	5′-TGCTGGAAATCAGTGGACCC-3′	5′-CGGGTTTTGATGGTAGGGCT-3′
GAPDH (Human)	5′-ATGGTGAAGGTCGGTGTGAA-3′	5′-TGGAAGATGGTGATGGGCTT-3′
GSDMD (Mouse)	5′-GATCAAGGAGGTAAGCGGCA-3′	5′-CACTCCGGTTCTGGTTCTGG-3′
GAPDH (Mouse)	5′-CCCTTAAGAGGGATGCTGCC-3′	5′-TACGGCCAAATCCGTTCACA-3′
